# Astrocyte metabolic programs diverge across primate evolution and are linked to species-specific neuronal development

**DOI:** 10.21203/rs.3.rs-11133580/v1

**Published:** 2026-10-14

**Authors:** Fred Gage, Sheila Steiner, Kaia Foster, Rebecca Chinn, Joshua Pratt, Sarah Fernandes, Amandeep Sharma, Renata Santos, Christian Metallo, Maria Marchetto

## Abstract

Humans are characterized by changes in neuronal development compared to non-human primates (NHPs), and metabolic changes are implicated in these species' differences. However, most research has focused on changes in the neurons and neural progenitor cells themselves, but astrocytes are crucial metabolic cells in the brain. Here, we identified a delayed onset of gliogenesis in human versus NHP organoid models. iPSC-derived astrocytes also revealed distinct metabolic profiles: NHP astrocytes exhibit increased serine and glycine synthesis, whereas human astrocytes show elevated lactate secretion, suggesting metabolic reprogramming of astrocytes across primate evolution. Astrocyte secretomes also differed, with human astrocyte secretomes enriched for axon-growth proteins. We then assessed the impact of these different species’ astrocyte secretomes on neuronal development. Conditioned media from NHPs as compared to humans led to increased electrophysiological activity and altered transcriptomic signatures of neuronal development in human neurons. Chemical PHGDH inhibition is consistent with a partial contribution of astrocytic serine/glycine metabolism to these species-specific effects. Together, these findings reveal a metabolic and secretory divergence between human and NHP astrocytes linked to species-specific neuronal development.

## Introduction

Astrocytes are fundamental to the function and development of neurons. They play important roles in metabolism, inflammatory response, neurotransmitter modulation, and synapse function (Barres 2008; Liddelow and Barres 2017; Allen and Lyons 2018). In an *in vitro* context astrocytes are critical to neuronal maturation, synaptically and electrophysiologically (Pfrieger and Barres 1997; Zheng et al. 2025; Hedegaard et al. 2020). Disruptions in astrocyte-neuron crosstalk play a central role in several neurodevelopmental disorders (Sejourne et al., 2024), highlighting the importance of astrocytes in proper neuronal development.

Metabolic genes are the locus of many genetic differences between humans and our closest non-human primate (NHP) relatives (Namba et al., 2021; Xing et al., 2024; Stepanova et al., 2021). These metabolic genes play a causative role in neural progenitor cell (NPC) proliferation. For example, ARHGAP11B, a human-specific gene, increases the amount of basal radial glia through modulating glutaminolysis, and the use of glutamine in the TCA cycle (Namba et al., 2020). Additionally, the humanized version of TKTL1 modulates basal radial glia populations by affecting fatty acid synthesis and the pentose phosphate pathway (Pinson et al., 2022).

Studies that compare mice and human cells demonstrate that manipulation of the metabolic features of NPCs impacts their development. For example, inducing human cells to perform more oxidative phosphorylation led to an increase in the rate of neuronal maturation (Iwata et al., 2023). Moreover, a preprint study demonstrated that increasing mitochondrial fatty acid oxidation in human organoids drives an increase in maturation rate (Iwata et al., 2025). Collectively, these data suggest that the metabolic properties of brain cells have changed significantly over the course of primate and mammalian evolution, and that metabolic changes can influence neurodevelopment.

However, these evolutionary metabolism studies have primarily focused on basal radial glia or NPCs. This focus makes sense as an increase in NPC proliferation is thought to underlie the unique expansion of human cortices (Kriegstein et al., 2006). However, astrocyte metabolism is critical to neuronal metabolism. For example, the astrocyte-neuron lactate shuttle has been implicated in neuronal activity and long-term memory formation (Suzuki et al., 2011). Inhibition of lactate transporters led to impairments in memory and synaptic plasticity in a mouse model (Suzuki et al., 2011). *In silico* studies indicated that the astrocyte-neuron lactate shuttle leads to greater theoretical maximum ATP production in neurons (Genc et al., 2011).

Finally, astrocytes produce several other building blocks for neurons, such as the amino acids and glia transmitters serine and glycine, fatty acids, and more complex lipids. For example, PHGDH, the enzyme that catalyzes the rate limiting step in serine synthesis, is primarily expressed in astrocytes in the brain (de Koning et al., 2003). Mutations in this gene are associated with microcephaly and cognitive symptoms (Poli et al., 2017; Jaeken et al., 1996; de Koning et al., 2003; Handzlik and Metallo 2023). Collectively, these results demonstrate how metabolic features of neurons are shaped by crosstalk with astrocytes, and several functions of neurons are dependent on the metabolites provided by astrocytes.

There is greater transcriptomic divergence between human and NHP astrocytes than neurons (Jorstad et al., 2023; Zintel et al., 2024). Human astrocytes, as compared to mouse astrocytes, show lower rates of oxidative phosphorylation and changes in the expression of metabolic genes (Li et al., 2021). *In vitro* astrocyte studies comparing human, chimp and rhesus show significant transcriptomic differences in metabolic genes (Ciuba et al., 2025; Zintel et al., 2024). However, despite this evidence of human-specific astrocyte features, research on astrocytes across primate evolution is limited compared to the research on NPCs and neurons.

Collectively, these data point to the hypothesis that astrocytes differ in their metabolic role across species and may play a role in species-specific features of neurodevelopment, perhaps in part through their role in metabolism. Our study addresses how astrocytes and their metabolism have changed in primate evolution and whether astrocytes play a role in modulating neurodevelopment in a species-specific manner.

First, we demonstrated successful generation of iPSC-derived astrocytes from several primate species. We also performed a transcriptomic characterization of these astrocytes. Next, we characterized the metabolic properties of these astrocytes to determine if astrocyte metabolism had changed significantly between humans and NHPs at a functional level. Finally, we tested whether treatment with different species' astrocyte conditioned media led to differences in neuronal development and function. By chemically inhibiting one crucial enzyme in the serine metabolism pathway, we demonstrate that some of the changes in neuronal function we observed with the astrocyte conditioned media were shaped by the differences in metabolic features of astrocytes between humans and NHPs.

## Results

### Neurons show changes in metabolic gene expression in humans versus NHPs

Metabolic genes show significant changes in regulation and coding sequence across the primate lineage, as has been established in the literature (Figure 1A) (Fedrigo et al., 2011; Pinson et al., 2022; Grossman et al., 2004; Burki et al., 2004; Xing et al., 2024; Ju et al., 2025; Stepanova et al., 2021; Florio et al., 2015; Namba et al., 2020; Duka et al., 2014). Re-analysis of sequencing data from Linker et al. (2022) showed differential expression of a key metabolic gene, LDHA, in human as opposed to NHP NPCs and neurons, suggesting we were able to recapitulate one of these metabolic differences in our *in vitro* system (Figure 1B). This result was validated by western blot in NPC derived neurons (2 human lines, 2 NHP lines) (Figure 1C) and has been observed in post-mortem tissue (Duka et al., 2014).

To determine whether human and NHP *in vitro* neurons had functional differences in metabolism, we collected NPC-derived neurons one week after the onset of differentiation and sorted them to obtain a pure neuronal population (Supplemental Figure 1A and 1B, Figure 1D). We then allowed the neurons to recover and performed [U-$^{13}C_6$] glucose tracing for 24 hours using three human and five NHP lines. There were no significant differences in labeling between human and NHP neurons for glycolytic or TCA cycle metabolites (Figure 1E). We also performed tracing with labeled lactate at the same time point in three human and four NHP lines (two gorillas and two chimpanzees). We found no differences in labeling or abundance of downstream metabolites (Supplemental Figure 1C-H). However, at three weeks post-differentiation, intracellular lactate showed significantly different labeling and a trend toward increased labeled abundance ($p = 0.067$), suggesting human neurons may take up more lactate than bonobo neurons. No differences appeared in any downstream metabolite (Supplemental Figure 1I-M). Since this three-week timepoint used only one line per species (due to limited cell yield after FACS sorting), we could not assess line-to-line variability. These results suggest that the metabolic gene expression differences may reflect species-specific differences in exogenous lactate uptake rather than broader glucose/lactate utilization, though further experiments are needed to conclude this definitively.

Interestingly, Schenz et al., 2021 demonstrated that extracellular lactate changes the expression of LDHA, suggesting that LDHA expression may be extrinsically regulated. However, the species differences in LDHA expression could also be driven by a regulatory change across species, as opposed to external stimuli.

**Gliogenesis and astrocyte maturation occur earlier in NHP organoids when compared to human organoids**

Neurons are dependent on astrocytes for many of their metabolic needs. Therefore, we investigated whether astrocytes may contribute to differences in brain metabolism across primate species. First, we sought to determine whether the timing of astrocyte development and gliogenesis differs across species. We generated forebrain organoids from three NHP and three human iPSC lines. We performed staining for astrocyte markers (early glia marker NFIA and mature marker S100B). Our data demonstrate that astrocytes arise in NHP organoids at earlier time points (Figures 1F and 1G). Given the difference in timing of gliogenesis in 3D cultures, we hypothesized that there may be differences in astrocytic provision of metabolites at early neurodevelopmental time points.

**Astrocytes show differences in metabolic gene expression in humans versus NHPs**

Given our hypothesis that metabolic differences between human and NHP astrocytes may exist, we generated a pure *in vitro* system to investigate the changes in astrocytes across species. We began by generating glial progenitors and astrocytes from a range of primate species *in vitro* using the protocol described in Santos et al. (2022) and Santos et al. (2017) (Figure 2A). We successfully made astrocytes from three human lines, two chimpanzee lines, two bonobo lines, one gorilla line and one rhesus macaque line. Astrocytes expressed key glial progenitor and astrocyte markers and showed a response to IL1-beta (Figures 2B- D).

To understand the differences in human versus NHP astrocytes, we began with a transcriptomic characterization of our human and NHP astrocytes at the glial progenitor cell (GPC) stage, three weeks and five weeks into *in vitro* maturation (Supplemental Figure 2A). Of note, we created a module score based on immature and mature astrocyte markers, as described in Zhang et al., 2016, and observed no significant differences in these module scores in humans versus NHPs (Supplemental Figure 2B and C). This lack of significant difference in maturation score was also observed in Ciuba et al., 2025. Although we cannot prove a negative, we believe that these data suggest that the differences in phenotypes we observed in these astrocytes are due to differences in astrocyte function at this developmental stage as opposed to differences in maturation state. We decided to focus on the GPC and five-week post differentiation stages for further downstream analyses. We ran differential expression analysis to determine which genes were differentially expressed between our human and NHP cell lines. We identified 615 differentially expressed genes at the GPC stage and 631 differentially expressed genes at the five-week-old astrocyte state (p-adjusted $< 0.05$) (Figure 2E-G). There were 175 genes that were significantly differentially expressed at both the GPC and five-week-old time point, and enrichment analysis primarily showed these genes were related to neuronal signaling (Figure 2G, Supplemental Figure 2D). Furthermore, there was a significant correlation between log fold change NHP/human as measured by DESEQ2 at the GPC state and astrocyte state, suggesting there were conserved differences in gene expression across species regardless of differentiation stage (Supplemental Figure 2E).

GSEA showed an enrichment for genes related to neuronal signaling and ligands at the GPC state (Figure 2H). At the five-week time point, we observed an upregulation in human astrocytes of genes involved in immune responses (Figure 2I, Supplemental Figure 2F). This trend in our data corresponds to previous literature that human astrocytes have a stronger response to inflammatory stimuli than mouse astrocytes (Li et al., 2021).

GSEA showed an upregulation of metabolic pathways in our NHP astrocytes at the five-week time point (Figure 2I, Supplemental Figure 2F). We specifically observed an enrichment of amino acid metabolism. These results support the changes in metabolism gene expression identified in Ciuba et al. (2025). Furthermore, we observed a general upregulation of glycine and serine metabolism (two pathways that were enriched) enzymes in the NHP astrocytes. We also observed a downregulation of aerobic glycolysis genes (most notably *HIF1A* and *SLC16A3*, a key aerobic glycolysis and lactate transport gene, respectively) in the NHP astrocytes compared to the human astrocytes at both time points (Figure 3A).

Because our *in vitro* astrocytes are immature compared to brain astrocytes, we turned to post-mortem data to validate the metabolic gene enrichment in human versus chimpanzee astrocytes. Using differentially expressed genes from the astrocyte cluster in Jorstad et al (2023), which performed post-mortem single nuclei transcriptomics on human and primates' middle temporal gyrus, we identified top pathways in MSigDB enrichment analysis (ranked by p-value). These pathways included oxidative phosphorylation, hypoxia (related to *HIF1A*), and glycolysis (Figure 3B). Average expression of *GLDC*, *PHGDH*, and *HIF1A* in the astrocyte clusters in this data showed a trend towards decreased expression of *PHGDH* and *GLDC* and increased expression of HIF1A in human compared to chimp, gorilla and rhesus, which parallels what we observed *in vitro* (Figure 3C). Statistical comparison was not possible, as Cytosplore Viewer reports only a single average expression value per species. These expression differences led us to hypothesize that these metabolic gene expression differences *in vitro* may also be relevant to astrocytes in the adult brains of humans and NHPs.

**NHP astrocytes show widespread metabolic restructuring compared to human astrocytes**

Next, we investigated whether the significant differences we observed in astrocyte metabolic gene expression translated to functional species differences in astrocyte metabolism. To quantify and compare metabolism between humans and other primates, we performed [U-$^{13}C_6$] glucose labeling experiments in our human and NHP lines at the one week and five-week time points (Figure 3D). At the five-week time point, we observed an increase in intracellular glycine and serine labeling from glucose in the NHP astrocytes (5 lines; bonobo, chimp, gorilla, rhesus) compared to the human astrocytes (2 lines) (Figure 3E and F; Supplemental Figure 3A-B). This experiment was repeated with three human lines and five NHP lines (two chimpanzees, one bonobo, one gorilla and one rhesus line) (Supplemental Figure 3C and D). We did not observe an increase in abundance of serine or glycine label in the media (Supplemental Figure 3E and F). We also observed a significant increase in glycine abundance as well as a trend towards increased serine abundance in the NHP astrocytes (Figure 3G and 3H; Supplemental Figure 3G-H). We interpreted these data as demonstrating an increase in *de-novo* synthesis from glucose of serine and glycine in the NHP astrocytes compared to the human astrocytes. This finding aligned with data from our transcriptomics assay in five-week-old astrocytes, which showed an upregulation of serine, glycine and threonine metabolism in NHP astrocytes (Figures 2I, 3A and 3C). This difference in serine and glycine labeling was not observed at our one-week time point, suggesting that this phenotype is found at more mature time points in astrocyte differentiation (Figures 3I and 3J, Supplemental Figure 3I-3J). This assay at the one-week time point was performed in two human lines, two chimps, one bonobo, one gorilla, and one rhesus line.

Human astrocytes showed a substantial increase in lactate release into the media as opposed to NHP astrocytes (Figure 3K). This observation correlated with our transcriptomic findings, which demonstrated an increase in the expression of *HIF1A* and *SLC16A3* (the main lactate exporter in astrocytes, MCT4) in human astrocytes.

Citrate abundance was also elevated in NHP astrocytes (Figure 3L) without a corresponding increase in labeling from glucose or glutamine (Figure 3M, Supplemental Figure 3M) and was accompanied by a trend toward increased fatty acid synthesis from glucose (Supplemental Figure 3K-L), suggesting possible diversion of citrate toward anabolic pathways rather than increased oxidative flux. We did not observe a significant increase in label or abundance of other TCA cycle related metabolites (Figure 3M), which is not inconsistent with our hypothesis that NHP astrocytes had increased labeling in anabolic pathways, supporting the synthesis of key metabolic precursors, as opposed to merely operating at a higher metabolic rate.

Collectively, these data suggested astrocytic metabolic reprogramming in humans compared to NHPs. Human astrocytes primarily release lactate whereas the NHP astrocytes perform more anabolism of key building blocks, which we hypothesize may be important for neuronal differentiation and maturation.

## Species Divergence in Astrocyte Secreted Proteins

To understand how astrocytes may differently affect neuronal features, we analyzed the proteomic secretome of human and NHP astrocytes. We performed proteomics on astrocyte conditioned media (ACM) from four NHP lines (bonobo, chimpanzee, gorilla, rhesus) and three human lines. PCA showed a clear separation between human and NHP samples (Supplemental Figure 4A), with similar numbers of proteins enriched in each species group (Supplemental Figure 4B). Human astrocytes secreted more of several axon guidance proteins such as *APOE* (Tensaouti et al., 2018; Yu et al., 2021), *NRCAM* (Sakurai et al, 2012) and *LAMA2* (Arreguin et al., 2020; Anderson et al., 2005) (Supplemental Figure 4C). GSEA GO revealed an upregulation in human ACM of neuronal branching and axon development terms (Supplemental Figure 4D). The leading edge genes from this GSEA revealed that several key genes involved in neuronal branching and axon development/guidance, such as *ROBO2* (Blockus et al., 2021; Zhang et al., 2012; Campbell et al., 2007), *SEMA3B* (Mohan et al., 2019), *DCC* (Horn et al., 2013), *EPHA7* (Clifford et al., 2014), and *LAMA2* (Arreguin et al., 2020) are found at higher amounts in human ACM (Figure 4A). Rather than promoting faster maturation, these proteins are associated with neuronal complexity/dendritic arborization, which led us to hypothesize that the human astrocytes may play a distinct role in shaping dendritic arborization when compared to the NHP astrocytes.

## Species-specific astrocyte-secreted factors alter neuronal differentiation-related gene expression and electrophysiological activity

Human cortical neurons undergo a prolonged developmental and maturation program (Marchetto et al., 2019; Somel et al., 2009; Schörnig et al., 2021; Pollen et al., 2019; Vanderhaeghen and Polleux 2023). We hypothesized that the NHP astrocyte-secreted factors may serve as a cell-extrinsic mechanism for changing neurodevelopmental trajectories and electrophysiological maturation.

To test this hypothesis, we cultured astrocytes from three human lines and four NHP lines (chimp, bonobo, gorilla, rhesus) in neuronal media for twenty-four hours, then fed it to human neural progenitor cells (NPCs). We measured changes in their transcriptome and electrical activity (Figure 4B). We collected the NPCs after conditioning for 1, 4, 7, or 14 days and performed differential expression and GO analysis between the human and NHP ACM treated neurons (p-adjusted $< 0.1$; see Methods). We observed an enrichment for terms related to neuronal differentiation, neuron fate commitment, and forebrain development (Figure 4C). We next identified ten genes that showed significant differential expression between human and NHP conditioned media conditions in at least three of the four-time points (Figure 4D and E). We observed an upregulation of *SMAD6* in neurons exposed to NHP ACM and an upregulation of *EBF2*, *VXN*, *NEUROD4*, *NEUROD1*, *NEUROG1* and *NEUROG2* in neurons exposed to human ACM. Several of the genes upregulated in the human ACM treated neurons are transiently expressed early markers of neuronal cell fate commitment and help establish and maintain developmental transcriptional programs (Chuang et al., 2011; Garcia-Dominguez et al., 2003; Lacomme et al, 2012; Bertrand et al., 2002; Moore et al., 2018). We hypothesize that their sustained expression may therefore reflect a more prolonged developmental state, consistent with the delayed maturation observed in human neurons in previous studies. Collectively, these data demonstrate a change in neuronal differentiation trajectories in those human neurons cultured with NHP versus human ACM and a change in the expression of key regulators of neuronal differentiation.

We next tested whether neurons exposed to conditioned media showed differences in electrical activity, a canonical metric of neuronal maturation. We treated two-week-old neurons from humans with conditioned media from one of two human lines or from one rhesus line for a total of three days (Figure 4F). Relative to the electrical activity in each well prior to the addition of ACM, there was an increase in the percent of active electrodes in those human neurons exposed to rhesus-conditioned media as opposed to human ACM (4G and 4H). We interpreted this as the faster electrophysiological maturation in the rhesus-conditioned media neurons.

Taken together, our transcriptomic, electrophysiological, and secretomic data support a model in which human and NHP astrocytes shape neurodevelopment trajectories differently. While NHP astrocytes appear to promote an increase in electrical activity, a key functional feature of a mature neuron; human astrocytes may preferentially provide cues that promote a more plastic, early neuron commitment transcriptional state.

## PHGDH inhibition and TGFβ1 shift NHP metabolism to a more human-like phenotype

We next investigated broader cellular pathways that may regulate the differences in metabolism we observed. We identified a general upregulation of TGFβ signaling pathways in human astrocytes in our transcriptomics dataset (Supplemental Figure 5A). TGFβ has been previously implicated as one of the regulators of metabolism (Liu et al., 2022). We added TGFβ1 to the media of astrocytes from one human line and from one NHP (chimpanzee) line and then performed a [U-$^{13}C_6$] glucose trace. In both the human and NHP lines, we observed a significant decrease in *de novo* serine and glycine synthesis and abundance (although glycine abundance was not significantly lower) (Supplemental Figure 5B-E).

Given the differences in serine synthesis we had observed, we investigated whether inhibiting PHGDH, using the small molecule NCT-503, could recapitulate our metabolic phenotype. We selected PHGDH because it commits glucose to serine/glycine synthesis and mutations in PHGDH are associated with neurological symptoms (Poli et al., 2017; Jaeken et al., 1996; de Koning et al., 2003; Handzlik and Metallo 2023; Eade et al., 2021). Using one chimpanzee and one human line, we performed a [U-$^{13}C_6$] glucose trace with NCT-503 treatment. We observed decreased *de novo* serine and glycine synthesis (Figures 5A and B) in both species. We also observed a significant decrease in serine and glycine abundance in both the human and chimpanzee line with PHGDH inhibition (Supplemental Figure 5F and G). Together, these data are consistent with

PHGDH-dependent serine/glycine metabolism contributing, at least in part, to the neuronal transcriptional response to ACM; given the reliance on a single pharmacological inhibitor and small sample size in these experiments, this should be considered a preliminary mechanistic link rather than established causation.

**PHGDH inhibition in astrocytes is associated with altered transcription of neuronal differentiation genes in neurons cultured with ACM**

Next, we tested how these differences in metabolism affected neurons cultured in ACM after the astrocytes had been treated with the PHGDH inhibitor. We used four conditions: chimp ACM ± PHGDH inhibitor, base neuronal media ± PHGDH inhibitor. Using DESEQ2, we identified genes that showed an interaction effect for PHGDH inhibition and ACM. This approach allowed us to identify genes that responded in neurons specifically to the impact of PHGDH inhibition on the astrocytes, not just the presence of the PHGDH inhibitor (Figure 5C).

We identified 301 differentially expressed genes (Figure 5D). When we inhibited PHGDH in the astrocytes, we observed an upregulation of several serine and glycine related genes in the neurons in the interaction effect, which is consistent with increased demand for cell-autonomous serine and glycine synthesis in neurons without provision by astrocytes (Figure 5E). Collectively, this result indicated that there was a remodeling of neuronal metabolic transcription programs in response to changes in the metabolism of astrocytes. GO enrichment of these genes included both metabolism-related terms and neuronal differentiation-related terms (Figure 5F).

We next tested whether the changes in genes that were differentially expressed in response to ACM from NHPs versus humans were mediated by PHGDH expression in the astrocytes. We created a module score of the genes significantly upregulated in NHP ACM versus human ACM. We observed higher expression of those genes in the chimpanzee ACM versus the no ACM condition. PHGDH inhibition in the chimpanzee astrocytes led to expression levels similar to the levels in neurons grown without ACM (Figure 5G). This result indicates that PHGDH inhibition in the astrocytes may ameliorate some of the effects of chimp ACM on neuronal gene expression and that the metabolic rearrangement of NHP astrocytes may contribute to the change in neuronal development trajectory we observed, though given the reliance on a single pharmacological inhibitor and cell line per species, this should be considered preliminary support for, rather than confirmation of, this mechanism.

## Discussion

Neuronal development is highly dependent on astrocytes; astrocytes assist with metabolism, synaptic pruning, and electrophysiological maturation (Barres et al., 2008). Therefore, we investigated whether there are species-specific differences in astrocytic function that may influence species-specific features of neuronal development.

In this study, we successfully generated iPSC-derived astrocytes from six NHP individuals, across four species, allowing us to study astrocyte function in a diverse cohort of NHPs. We also demonstrated a decrease in the expression of metabolic genes in human versus NHP astrocytes. We revealed a human-specific specialization in lactate release, whereas NHP astrocytes were characterized by an increase in serine and glycine synthesis. We found that these astrocyte-level differences are associated with functional consequences for neurons:

exposure to NHP ACM was associated with increased electrophysiological activity and altered expression of neuronal differentiation genes relative to human ACM.

Comparisons between mouse and human cells have previously identified changes in metabolic function. Li et al. (2021) demonstrated a decreased OCR/ECAR ratio in human astrocytes compared to mouse astrocytes. Furthermore, a comparison of mouse and human immune cells showed changes in serine metabolism in activated NK cells (Li et al., 2025). However, to our knowledge, functional metabolic studies have not been done in human versus NHP astrocytes.

In our data, we observed a human-specific increase in lactate release in astrocytes. These data could indicate a species-specific divergence in the astrocyte-neuron lactate shuttle. Neurons can use the lactate as an energy source for the TCA cycle (Karagiannis et al., 2021; Wyss et al., 2011). An *in silico* study suggested that lactate provision by astrocytes increases neuronal ATP, which could help support a more energetically demanding neuron (Genc et al., 2011). Human neurons are larger and more morphologically complex cells (Libe-Phillipot et al., 2024; Lindhout et al., 2024) which may require more ATP to support action potentials (Sengupta et al., 2013). However, the increases in serine and glycine synthesis and citrate abundance suggest that NHP astrocytes are biosynthetically more active, potentially synthesizing more proteins or lipids, which may be helpful in providing building blocks for developing neurons.

Mutations in PHGDH, the rate limiting enzyme in serine synthesis, can lead to neurological syndromes and changes in neuronal differentiation (Poli et al., 2017; Jaeken et al., 1996; de Koning et al., 2003; Handzlik and Metallo 2023). Furthermore, serine is a critical component of sphingolipids. Sphingolipids, particularly gangliosides, are essential components of neuronal membranes that regulate membrane integrity, signaling, and developmental processes including neuronal maturation. Disruption of sphingolipid metabolism or synthesis can impair neuronal development and contribute to neurological disease (Schwarz et al., 1995; Olsen & Færgeman, 2017; Chinnappa et al., 2025). An increase in serine synthesis could be coordinated with an increase in sphingolipid synthesis, which could have downstream effects on neurons.

Conditioned media from NHP astrocytes led to an increase in electrophysiological activity and changes in the expression of neuron differentiation and maturation genes in human neurons. Human neurons take longer to reach terminal and electrophysiological maturation than NHP neurons (Marchetto et al., 2019; Somel et al., 2009; Schörnig et al., 2021; Pollen et al., 2019; Vanderhaeghen and Polleux 2023). Human neurons eventually reach a more complex state, characterized by increased soma size, more complex branching, and increased synapse numbers (Lindhout et al., 2024). We hypothesize based on the data presented here that the NHP astrocytes may contribute to the earlier electrophysiological maturation of neurons. However, the human astrocytes release more axonogenic proteins (as observed in our secretomics data), which we hypothesize may contribute to the increased neuronal branching reported in human neurons in other studies. These data provide hints towards different roles for human and NHP astrocytes in neurodevelopmental support. However, follow up studies at later time points to determine whether neurons exposed to human ACM show an eventual increase in complexity would be necessary to conclusively determine this.

Finally, inhibition of the key step in serine synthesis reduced serine and glycine synthesis in NHP astrocytes. Treating human neurons with conditioned media from PHGDH inhibitor-treated NHP astrocytes led to downregulation of neuronal differentiation genes. Together, these data are consistent with a partial contribution of NHP astrocytic serine synthesis and related metabolic pathways to the species-specific neuronal transcriptional responses we observed, though this evidence is based on pharmacological inhibition in a limited number of cell lines and should be interpreted as preliminary support for, rather than confirmation of, a causal metabolic mechanism.

**Limitations**

All the experiments in this paper were performed using *in vitro* models of astrocyte development. *In vitro* astrocytes are less mature, and the metabolic environment of these cells differs from the *in vivo* environment. Astrocytes *in vitro* more closely resemble fetal astrocytes (Zhang et al., 2016; Ciuba et al., 2025), suggesting that the species differences we observed may not reflect differences in adult astrocytes. In partial support of the relevance of our findings, we have reanalyzed scRNA-sequencing from post-mortem human and chimpanzee astrocytes (from Jorstad et al., 2023) and we observed an enrichment for transcriptomic changes in metabolism. However, it is not experimentally feasible to perform metabolic studies on astrocytes from human and chimpanzee adult tissue.

Determining functional characterization of neuronal maturation is challenging, and we assessed it with only two metrics (electrophysiology and transcriptomics). Other metrics of neuronal maturation would be relevant for future study.

Our downstream assays demonstrate a partial amelioration of the effect of chimpanzee astrocytes on neuronal maturation with a PHGDH inhibitor; these data suggest that part of the impact of conditioned media was metabolically mediated, but we believe that it is likely mediated by several features of the secretome, including both metabolic and proteomic components in the media.

The PHGDH chemical inhibitor we used, NCT-503, can sometimes have other metabolic effects (Arlt et al., 2021). This raises the possibility that some of the changes we observed in neuronal transcription in neurons exposed to astrocytes treated with NCT-503 may be due to differences in metabolic pathways other than serine/glycine synthesis. Finally, we performed our PHGDH experiments in one human and one chimp line, which has been standard in the field for cross-species metabolic experiments (Iwata et al., 2023), but we cannot distinguish a true species-level effect from line-specific variability.

## Methods

### ***Cell Culture***

*Stem Cell Lines*

Fibroblasts from four neurotypical humans (WT126, WT33, 14961 and MS-2C), two chimpanzees (PR00818 and PR01029), two bonobos (PR01086 and AG05253), two gorilla (PR00075 and PR00053), and one rhesus (A1) were reprogrammed to iPSCs as previously described (Linker et al., 2022, Marchetto et al., 2013, Marchetto et al., 2010, Marchetto et al., 2019, Mertens et al., 2015). Orangutan iPS were generously provided by the Haussler/Salama labs (JOS3C1). Established iPS were kept in feeder-free conditions and passaged with Gentle or ReLeSR. Rhesus iPS were maintained in MEF conditioned media supplemented with FGF2. NPC lines were derived as previously described in Linker et al., 2022. The use of fibroblasts from chimpanzee, bonobo and gorilla were approved by the US Fish and Wildlife service, under permit MA206206. Protocols describing the use of iPSCs were previously approved by the University of California, San Diego, and the Salk Institute IRB.

*NPC to Neuron Cell Culture*

NPCs were thawed onto Matrigel or PLO/Lam plates and grown in DMEM/F12 + Glutamax + N2 + B27, with 20 ng/mL FGF2 added fresh each feed. They were split onto PLO/Lam plates and switched to neuronal differentiation media, which is as follows: DMEM/F12 + Glutamax + N2 + B27 + 20 ng/mL BDNF + 20 ng/mL GDNF + 500 ug/mL cAMP + 0.2 nM Ascorbic Acid + 1 $\mu$g/mL Laminin.

*Generation of Glial Progenitor Cells from iPS Cells*

To generate glial progenitor cells, we followed the protocol as described in Santos et al. (2022) and Santos et al. (2017). In brief, we generated EBs from iPS using collagenase to generate clumps of iPS and placed them into ultra-low attachment 6 well plates. The next day, we changed the media to ScienCell Astro Media + 2% FBS and 500 ng/mL of Noggin, and 10 ng/mL of PDGF-AA. D15 after EB generation we switched the media to ScienCell Astro Media + 2% FBS + PDGF-AA at 10 ng/mL. On day 21 we dissociated the EBs using a Papain + DNase solution and plated the dissociated cells in Science Cell Astro Media + 2% FBS + 20 ng/mL FGF2 + 20 ng/mL EGF, 1 $\mu$g/mL Laminin and 10 $\mu$M Rock-I on Plo/Lam coated plates. The next day, cells were changed to ScienCell Astro Media + 2% FBS + 20 ng/mL FGF2 + 20 ng /mL EGF and were maintained in this media on Plo/Lam plates.

*Generation of Astrocytes from Glial Progenitor Cells*

To generate astrocytes from GPCs, we changed the media to DMEM/F12 + Glutamax + N2 + B27 – vit A + 10% FBS. On D15, we used Accutase to split cells onto uncoated plates to remove undifferentiated progenitor cells. At five weeks post-onset of differentiation, cells were considered mature.

*IL-1$\beta$ Treatment*

At the five-week timepoint, astrocytes were treated with 10 ng/mL of IL-1β for five hours. They, along with a neighboring well from the same line that was not treated, were collected in Trizol for RNA-sequencing.

*Organoid Culture*

Organoid protocol is as described in Qian et al., 2018. iPS from each species were split onto MEFs. iPS + MEF cultures were fed hES media (DMEM/F12 + 20% KOSR + 1% Glutamax + 1% NEAA + 0.1% 2-Mercaptoethanol + 1% Pen Strep). Once iPS were confluent, they were split with collagenase, transferred to a 15 mL conical tube and allowed to gravity sedimentate in DMEM. They were replated in 10 cm ULA plates and fed hES media + 1:1000 rock inhibitor and 4 ng/mL of FGF2. For Days 1 – 4 EBs were fed hES + 1:5000 A83 (Tocris #2939, stock concentration 10 mM) + 1:5000 Dorsomorphin (Torcis #3093, stock concentration 10 mM). From Days 5 – 7 (or until embedding) cells were fed Forebrain Second Medium (DMEM/F12 + 1% N2 + 1% Glutamax + 1% NEAA + 1% Penn Strep + 1 uM CHIR (Tocris #4423, stock concentration 20 mM) + 1 $\mu$M SB431542 (Torcis #1614, stock concentration 50 mM)).

Organoids with smooth edges were embedded in Matrigel at about the 7-day time point. Cells were fed Forebrain Second Medium from days 7 – 14.On Day 14, organoids were removed from the Matrigel using a surgical knife. Organoids were switched to forebrain third medium (DMEM/F12 + 1% N2 + 2% B27 + 1% Glutamax + 1% NEAA + 1x 2-Mercaptoethanol + 1% Pen-Strep + 2.5 $\mu$g/mL Insulin (Millipore #I9278). At day 70, cells were switched to forebrain fourth medium (Neurobasal Media + 2% B27 + 1% Glutamax + 1% NEAA + 1% Pen/Strep + 0.2 mM Ascorbic Acid (Stemcell Tech #72132) + 0.5 mM cAMP (Tocris #1141) + 20 ng/mL BDNF (R&D #248-BDB) + 20 ng/ml GDNF (R&D #212-GD).

***<u>Metabolic Tracing</u>***

*NPC to Neuron Metabolic Tracing*

NPC-derived neurons were lifted at one-week post onset of differentiation (as described above) with Accutase. Cells were stained with CD44-GFP and PSA-NCAM-PE for 45 minutes and a negative control was separated. Cells were filtered to remove clumps and FACS sorted to select for CD44-, PSA-NCAM+ cells. They were collected, spun down and replated at 200,000 cells per well of a 12 well on Plo/Lam coated plates. Cells were allowed to recover for several days and then treated with tracing media which consisted of: DMEM/F12 without glucose, 17.5 mM labeled glucose, 2 mM lactate, 3.5 mM glutamine, 1:50 B27 + VitA, 1:100 N2, 20 ng/mL BDNF (R&D #248-BDB), 20 ng/mL GDNF (R&D #212-GD), 500 $\mu$g/mL cAMP (Tocris #1141), 0.2 nM Ascorbic Acid (Stemcell Tech #72132), 1 $\mu$g/mL Laminin. For the 1 week and three week lactate trace, cells were fed: DMEM/F12 + 1:50 B27 + VitA, 1:100 N2, 20 ng/mL BDNF (R&D #248-BDB), 20 ng/mL GDNF (R&D #212-GD), 500 $\mu$g/mL cAMP (Tocris #1141), 0.2 nM Ascorbic Acid (Stemcell Tech #72132), 1 $\mu$g/mL Laminin + 3.5 mM Glutamine + 17.5 mM Glucose + 2 mM labeled lactate. After 24 hours, cells were collected as described for astrocyte tracing.

*Astrocyte Tracing*

At 5 weeks post-differentiation, astrocytes were plated at 200,000 astrocytes per well of a 12 well plate in three technical replicates per line. Two days post-plating, astrocytes were fed tracing media. Tracing media consists of DMEM/F12 without glucose, 2 mM glutamax, 1:100 N2, 1:50 B27, 1% FBS and [U-$^{13}C_6$] glucose for the glucose trace, and for the glutamine trace consisted of DMEM/F12, 2 mM [U-$^{13}C_5$] glutamine, 1:100 N2, 1:50 B27, and 1% FBS. Cells were collected as follows 48 hours following trace media addition:

First, media was collected from cells and placed into 1.5 mL Eppendorf tubes and spun down for 10 minutes at 4 degrees Celsius at max speed. Meanwhile, 1 mL 0.9% saline solution was added to each well of the twelve wells, and then removed as a rinse step. Quickly, 700 $\mu$L of cold 60/40 MeOH/H2O was added to each of the twelve wells. 50 $\mu$L of 100 $\mu$M norvaline and 2.5 $\mu$L of a fatty acid standard were added to each well. The cells were then scraped off the plate and collected in 1.5 mL Safe-Lock Eppendorf tubes. Cells were vortexed for 5 minutes. Then 75.2 $\mu$L of each sample was added to a clear 96 well plate to dry out for downstream BCA protein quantification. 500 $\mu$L of chloroform was added to each tube, followed by a 5-minute vortex and a 5-minute spin down at max speed at 4 degrees Celsius. 300 $\mu$L of the top phase (termed the polar phase) of each Eppendorf tube was added to a mass spectrometry vial and placed open in a Centrivap at 4 degrees to dry down overnight, after which it is frozen at -80 degrees Celsius. 500 $\mu$L of the bottom layer was extracted and placed into another 1.5 mL Eppendorf. This liquid was dried out under air and stored at -80 degrees Celsius.

Frozen samples were run on the mass spectrometer and analyzed using the protocol described in Green et al., 2024. Abundance values were normalized to a BCA performed on the dried down samples described above. Values for technical replicates for the same line were averaged together for downstream analyses for those plots that show humans vs non-human primates.

To analyze fatty acids, first the samples were thawed and redried under air for 10 - 15 minutes. 500 $\mu$L of 2% $H_2S0_4$ in MEOH was added to each sample, and then the samples were incubated at 50 degrees Celsius for two hours. 100 $\mu$L of saturated salt solution was added. 500 $\mu$L MS-grade hexane was added, and samples were vortexed. After the samples settled, the top layer was placed into a new Eppendorf tube. This was then dried under air, and resuspended in 75 $\mu$L of hexane, and vortexed for 10 seconds. Samples were transferred to a glass GC-MS vial with a glass insert and run on GC-MS.

*GC-MS*

Dried polar and nonpolar metabolites were processed for gas chromatography–mass spectrometry (GC-MS) as described previously by Cordes and Metallo (2021). Briefly, polar and interface metabolites were derivatized using a Gerstel MultiPurpose Sampler (MPS 2XL).

Methoxime–tert-butyldimethylchlorosilane (tBDMS) derivatives were formed by addition of 15 μL of 2% (w/v) methoxylamine hydrochloride (MP Biomedicals, Solon, OH) in pyridine and incubated at 45°C for 60 minutes. Samples were then silylated by addition of 15 μL of N-tert-butyldimethylsily-N-methyltrifluoroacetamide (MTBSTFA) with 1% tBDMS (Regis Technologies, Morton Grove, IL) and incubated at 45°C for 30 min. Derivatized polar samples were injected into a GC-MS using a DB-35MS column (30 m x 0.25 mm i.d.; Agilent J&W Scientific, Santa Clara, CA) installed in an Agilent 7890B GC system integrated with an Agilent 5977a MS. For polar metabolites, samples were injected at a GC oven temperature of 100°C which was held for 1 min before ramping to 255°C at 3.5°C/min and then to 320°C at 15°C/min and held for 3 min. For separation of the biomass metabolites the GC oven was held at 80°C for 6 min after injection, increased to 300°C at 6°C/min and held for 10 min, and finally increased to 325°C at 10°C/min and held for 4 min. Electron impact ionization was performed with the MS scanning over the range of 100 to 650 mass/charge ratio (m/z) for polar metabolites and 70-850 m/z for interface metabolites.

Nonpolar metabolites were saponified and transesterified to fatty acid methyl esters (FAMEs) by adding 500 µL of 2% $H_2SO_4$ in methanol to the dried nonpolar layer and heating at 50°C for 2 hours. One hundred uL of saturated NaCl and 500 uL of hexane were added, samples were vortexed, and the upper hexane phase was collected and transferred into a GC-MS vial. FAMEs were analyzed using a FAME Select column (100 m x 0.25 mm i.d.; Agilent J&W Scientific, Santa Clara, CA) installed in an Agilent 7890A GC system interfaced with an Agilent 5975 C MS using the following temperature program: 80C initial, increased by 20C/min to 170C, increase by 1C min/ to 204C, then 20C/min to 250C and hold for 10 min. The percent isotopologue distribution of each metabolite was determined and corrected for natural abundance using in-house algorithms that integrate the metabolite fragment ions and correct for natural isotope abundances.

*Astrocyte Tracing with PHGDHi and* TGFβ*1*

Cells were plated at a density of 400k cells/well of a 12 well. One day before trace media was added, PHGDH inhibitor NCT-503 (CAS 1916571-90-8) at concentration 5 uM or TGFβ1 (R&D #240-B/CF) at 1:5000 (stock concentration 50 ug/mL) was added to cells. Then 13C glucose tracing media was added to astrocytes along with fresh PHGDHi or TGFβ1 and cells were collected 48 hours later as described above and run on the mass spectrometer as described above.

## ***Sequencing***

*RNA seq Processing of Astrocytes and GPCs*

To collect RNA, cells were washed with PBS and collected in 1 mL of Trizol at the GPC, three-week and five-week time points. To extract RNA, 200 $\mu$L of chloroform were added to Trizol samples. Samples were put on ice for 5 minutes, then spun down at 12000 RCF for 15 minutes. Then the clear top layer was extracted, and an equal volume of 100% EtOH was added. RNA was extracted and purified using the RNA Clean and Concentrator Kit -5 (Zymo, Catalog # R1013). RNA was quantified using Qubit, and quality of extraction was assessed with TapeStation. Samples were submitted for sequencing at the Salk Integrative Genomics Core. Bonobo AG and Gorilla 75 GPCs and five-week astrocytes were collected and sequenced later. RNA was sequenced on a Novaseq X Plus with 20+ million reads/sample.

*RNA seq Analysis of Astrocytes and GPCs*

RNA data were aligned to the genome of the appropriate species (GCF_029289425.2, GCF_028858775.2, GCF_029281585.2,CHM13v2.0, or GCF_049350105.2). Nextflow was used for alignment, processing and QC with the following parameters: --trimmer fastp --extra_fastp_args=--trim_poly_g --pseudo_aligner kallisto --skip_alignment true.

Data was loaded into R and non-orthologous genes or genes that had one-to-many relationships were removed, except in the case of specific genes of interest (Figure 2D). Low count and ribosomal and mitochondrial genes were removed. Raw counts were put into DESEQ2 and the formula ~batch + nhp_human was used for GPC and 5-week analyses, and a p-value cutoff of 0.05 was used. Technical replicates were collapsed using DESEQ2 before differential expression analysis. Combat was used to perform batch correction for visualization purposes and PCA. P-values for expression (Figure 2D) were pulled from DESEQ2. GSEA was performed using KEGG with the gseKEGG function, p-value cutoff of 0.1, and minGSSize of 5. Log fold change values were derived from DESEQ2 output.

*RNA seq Processing of CM Experiments*

Three human astrocyte lines and four non-human primate astrocyte lines that were five weeks differentiated were split at the same cell density for each line. They were then transferred to neuronal media, and media was collected every 24 hours for a week from each line. Media was filtered to remove dead cells and frozen. A human NPC line (WT126) was thawed and was treated with neuronal media from one of the astrocyte lines. Neurons were collected either after one day, four-days, seven days, or fourteen days in astrocyte-conditioned neuronal maturation media. A control for each time point was also collected in neuronal media without any astrocyte conditioning. RNA was extracted and purified as described for Astrocyte and GPC RNA collection. RNA was sequenced on a Novaseq X Plus with 30 - 40 M reads per sample.

*RNA seq Analysis of CM Experiments*

RNA data were aligned, processed and QC was performed using nextflow with the hg38 genome and kallisto. FASTP was used for trimming with the –trim_poly_q option. Data was loaded into R and analyzed using edgeR with the formula ~batch + nhp_human_CM. Analysis was performed at each time point (d1, 4, 7 or 14 in conditioned media). P-value cutoff of 0.05 was used. Technical replicates were collapsed. LFC values were pulled from edgeR comparisons. GO analysis was performed using enrichGO with p-value cutoff of 0.1. For PCA plot/visualization purposes, and not differential expression analysis. Combat was used to perform batch correction for two samples that were collected at a different time.

*RNA seq Processing of PHGDHi*

One non-human primate astrocyte line (Chimp 818) that was five weeks differentiated was split at the same cell density for each line. Astrocytes were treated with a PHGDH inhibitor (NCT-503; HY-101966) for 24 hours to acclimate cells. Astrocytes were then transferred to neuronal media with a PHGDH inhibitor, and media was collected after 24 hours. Media was filtered to remove dead cells and debris. A human NPC line (WT126) was thawed, and cells were treated with neuronal media from one of the astrocyte lines. Neurons were collected after one day in astrocyte-conditioned + PHGDH inhibitor neuronal maturation media. Three controls were used; a control was collected in standard Chimp 818 astrocyte conditioned media without PHGDH inhibition, another was collected in neuronal differentiation media without astrocyte conditioning, and a third was collected from cells in neuronal differentiation media with the same concentration of the PHGDH inhibitor as was applied to the astrocytes. The third control's media was placed in the incubator in an empty well for 24 hours to ensure similar degradation of the PHGDH inhibitor compound as was present for the astrocytes. RNA was extracted and purified as described for Astrocyte and GPC RNA collection. Different wells of NPC derived neurons were treated as replicates. Three replicates were collected for the control chimp conditioned media and PHGDHi chimp conditioned media condition and two were collected for the other two controls. RNA was sequenced using Plasmidsaurus, with 10 million reads per sample.

*RNA seq Analysis of PHGDHi*

RNA data were aligned, processed and QC was performed using nextflow with the hg38 genome and kallisto. FASTP was used for trimming with the –trim_poly_q option. Data was loaded into R and EdgeR was performed with the formula ~treatment + conditioned media + treatment: conditioned media. GO analysis was performed by using the function enrichGO. Genes that were significantly upregulated in the non-human primate astrocyte conditioned media versus human astrocyte conditioned media at the four day time point from the initial experiments were selected and their VST normalized counts were averaged together to create a module score for each sample. Each dot represents a replicate (different wells of NPCs). T-tests were performed in ggplot2 to compare the module scores.

***Proteomics***

Five-week-old astrocytes were fed phenol-free DMEM/F12 + glutamax for 72 hours. Media was collected, frozen at -80, and given to the proteomics core for processing.

*Proteomics Methods*

Conditioned media were mixed 1:1 with a buffer containing 8 M GuHCl, 10 mM TCEP, and 40 mM CAA in 100 mM TEAB and incubated for 15 mins in the dark at 95 C to promote reduction and alkylation of disulfide bonds. The samples were then chloroform-methanol precipitated and briefly air dried. Protein precipitates were resuspended in 800 mM GuHCl in 100 mM TEAB. Proteins were digested with trypsin (1:30) overnight at 37 C. On the following day the digestion was stopped with 1% TFA, and the peptides were desalted with Empore SDB-XC StageTips (CDS Analytical). Protein amounts were normalized via a BCA assay (Pierce).

After drying in a SpeedVac, the peptides were resuspended in 0.1% FA in 5% ACN and injected into a Vanquish Neo UHPLC coupled to an Astral mass spectrometer (Thermo Fisher). Peptides were separated on a 50 cm Acclaim PepMap C18 UHPLC column (Thermo Fisher) at a flow rate of 300 nL/min. The mobile phases were 0.1% FA in water (A) and 0.1% FA in 80% ACN (B). The separation conditions were 6-12% B over 2 min, 12-28% B over 16 min, and 28-42% B over 7 min. The column was then washed at 99% B and re-equilibrated for the next sample. The mass spectrometer was operated in positive mode for data independent acquisition with a Nanospray Flex source for ionization. Full MS scans from 380-980 m/z were acquired in the Orbitrap at 240k resolution with a max injection time of 3 ms. Data independent scans were acquired in the Astral using a 2 Th isolation window, a custom AGC target of 300%, and a max injection time of 3 ms. Precursors were fragmented via HCD at 25% NCE and scans were collected from 150-2000 m/z.

LC-MS data were analyzed with CHIMERYS 4 in ProteomeDiscoverer v3.2 (Thermo Fisher) against the Uniprot Homo sapiens, Pan paniscus, Pan troglodytes, Gorilla gorilla, or Macaca mulatta reference proteome. Search settings included a mass tolerance of 10 ppm, a minimum peptide length of 7, and a maximum peptide length of 30. Full tryptic digestion was specified with a maximum of 2 missed cleavages. Modifications included fixed cysteine carbamidomethylation and variable methionine oxidation. Data was filtered at a 1% FDR at both the PSM and protein level.

Quantitation was done with the MS2 Apex strategy which uses the highest CHIMERYS coefficients from each precursor (after the 1% PSM FDR has been applied) from consecutive MS2 scans. Peptide abundances were normalized to total peptide amount, and protein abundances were calculated as the summed abundances of the connected peptides. The results were further filtered to remove proteins with only one peptide.

*Proteomics Analysis*

Proteins were summed across gene name. MS stats was run on the normalized abundance values to generate p-values comparing human and non-human primate samples. P-value cutoff of 0.05 was used. Technical replicates were entered as such in the MSstats dataProcess function. Proteins with a missingPercentage > 0.3 were not used in GSEA or the heatmap as shown in Supplemental Figure 4. GSEA GO analysis was performed on the filtered data.

## ***Western Blot of LDHA/LDHB***

Cells were collected at the NPC state, three days post-differentiation and seven-days post differentiation from two human lines, one chimpanzee line and one gorilla line. Cell pellets were lysed with RIPA buffer and quantified using a BCA. Sample buffer was created as a master mix of BME and LDS buffer. Buffer, water and sample were mixed to a total volume of 25 $\mu$L to have an equal amount of protein between samples. Proteins were denatured at 70C for 10 minutes and then spun down. Running buffer was made by mixing 950 mL H20 with 50 mL Bolt MOPS SDS running buffer. Samples were loaded into a 4-12% Bolt BT Plus gel. Gel was run at 100 v for the first 15 minutes and then 150v until band reached the bottom of the gel. Gel was transferred using an IBlot and blocked with Odyssey blocking buffer for 1 hour at room temperature. Samples were then incubated overnight at 4 degrees Celsius in primary antibody solution. The next day, samples were washed in 0.1% PBST and shook with secondary antibody solution at room temperature for 1 hour. Blots were washed 5 times in 0.1% PBST and imaged with the Odyssey CLX. We used the LDHA antibody #3582T from Cell Signaling Technology and the LDHB antibody sc-100775 from Santa Cruz Biotechnology.

## ***MEA of Human Neurons***

NPCs were differentiated to neurons as described above for two weeks. They were then split with Accutase and plated onto Plo/Lam coated Maxwell 6 well plates at a density of 400k neurons/well. Neurons were allowed to recover for several days, and then a baseline recording was measured using the Maxwell. We used a firing rate threshold of 0.02 and an amplitude threshold of 20 microvolts. We recorded an activity scan for 17 minutes with 30 seconds per configuration on the full well. Then astrocyte conditioned media (collected as described above) from human and rhesus astrocytes after 24 hours of conditioning and filtering to remove cells, was added to the wells. Conditioned media was changed daily, and were recorded for several days after the baseline recording.

Wells with neurons that came off in the recording were removed, and those whose maximum % active area was less than 1.5%. Each well's percent active area was normalized to its percent active area in the baseline recording. A t-test was performed comparing human and rhesus astrocyte conditioned media at each time point.

***Staining and Imaging of Astrocytes***

To assess purity of astrocyte populations, cells were plated on 8 well slides at about 25,000 cells per well. Cells were fixed with 4% Formaldehyde solution, and then washed with PBS 3x. Cells were then treated with 0.5% Triton X-100 in PBS for one hour at room temperature. Then cells were incubated with primary antibodies in 5% horse serum in PBS overnight at 4 degrees C. The next day, cells were incubated in secondary antibody in 5% horse serum in PBS for one hour at room temperature. Cells were counterstained with DAPI for 5 minutes and then immunomount was used to mount the cells with glass slides.

For astrocytes, the following antibodies were used: rabbit anti-S100b (1:500, catalog # ab52642), mouse anti-EAAT2 (1:100, catalog # sc-365634), and chicken anti-GFAP (1:500, catalog # ab5541). The Bonobo AG line was stained at a different time than the other astrocyte lines.

Images of astrocytes were taken using an Airyscan 880 at 20x. Images were standardized across lines for laser intensity and gain to make them comparable, except for Bonobo AG because it was stained at a different time, and was brighter than the other lines, despite the same antibody concentrations.

Purity analysis for astrocytes was performed on representative 20x images. The image analysis was performed blinded to which antibody was captured and which line was being analyzed (except for Bonobo AG). Image fluorescence channels were split, and each channel was standardized to the same color balance threshold across images. With only the DAPI channel on, the images were divided into quadrants and the locations of ~20 DAPI-stained nuclei were marked in each quadrant of the image. The exact number of DAPI nuclei marked was recorded for each quadrant. The DAPI channel was then turned off and another antibody channel was turned on. The number of marked spots with visible fluorescence was recorded. This was repeated for each channel, and the percentage of cells positive for each antibody was calculated by dividing the number of marks positive for fluorescence in that channel over the total number of marks.

***Staining and Imaging of Organoids***

At each time point, five organoids were removed from the culture. They were washed in PBS 3x, fixed in PFA for 45 minutes, then washed in PO4 3x. They were then kept in 15% sucrose overnight on the shaker, then 30% sucrose overnight on the shaker. The organoids were put into molds and frozen on dry ice in TFM. They were then stored in the -80$^{o}$C freezer.

Organoid blocks were then sectioned using a Cryostat at sections of 20 microns and mounted onto slides. Slides were frozen at -80$^{o}$C until ready to stain. To stain, each slide border was traced with a pap-pen and then inserted into a coplin jar and washed with PBS for 5 minutes. Slides were permeabilized in a 0.1% Triton X PBS solution for 10 minutes on the shaker. 500 $\mu$L of blocking buffer (5% NDS in 0.1% triton solution) were added per slide for one hour in a humidity chamber at room temperature. Primary antibody diluted in blocking buffer was added at 250 $\mu$L/slide and incubated in the humidity chamber on a shaker at 4 degrees Celsius overnight.

Slides were washed in PBT (0.05% Tween in PBS) 3x for five minutes each. Secondary antibody (1:200) diluted in blocking buffer was added to slides at 250 $\mu$L/slide and incubated at 1 hour at room temperature in the humidity chamber. Slides were washed three times in PBT for 5 minutes/wash. Slides were then counterstained with DAPI (1:1000 in PBT) for five minutes at room temperature in the humidity chamber, and then washed 2x with PBS for five minutes per wash. Three drops of immunomount were added per slide, and the coverslip was mounted.

Primary antibody used were S100B (catalog # NBP2-45257, concentration 1:1000) and NFIA (catalog # 3937, active motif, concentration 1:500). Imaging was performed on an Zeiss Axioscan 7 at 20x. Imaging settings were kept consistent across all images to ensure they are comparable. In order to quantify the percent area, the pixel classifier in QuPath 6.0 was used, and several images were used as training data. To quantify the percent positive cells, we used the cell detector method in QuPath.

## ***Statistical Methods***

Statistical analysis was performed in R or Graphpad Prism, as stated in the corresponding figure captions. For metabolomics, staining and MEA analysis, unless otherwise noted, a student's t-test was performed. For proteomics and RNA-sequencing data, DESeq2, edgeR or Msstats were used, as described above with appropriate p-value corrections performed. Each corresponding figure caption contains the formula used to perform differential expression as well. Adjusted p-value of 0.05 was used unless otherwise stated. Throughout, "N" refers to biological replicates, meaning lines derived from different individuals, unless otherwise stated. For most cases, technical replicates are averaged. The exception is Figure 5 and Supplemental Figure 5, which contain data from the PHGDHi experiment, in which we used separate wells of the same line as our replicates and the MEA experiments where each well is a different replicate because we only have one rhesus line in the lab. Exact test, n, and what n signifies for each experiment can be found in the corresponding figure legend. All NHPs are grouped together in the metabolomics analyses, but breakdowns by line can be found for some experiments in the supplemental figures.

## ***AI Usage***

LLMs were used to assist with generating plotting code in R for figures, and for language refinement and conciseness.

**Data and materials availability:** All raw mass spectrometry data will be uploaded to Metabolomics Workbench. RNA-seq datasets and counts files will be available through GEO.

**Declaration of Interests**

The authors declare no competing interests.

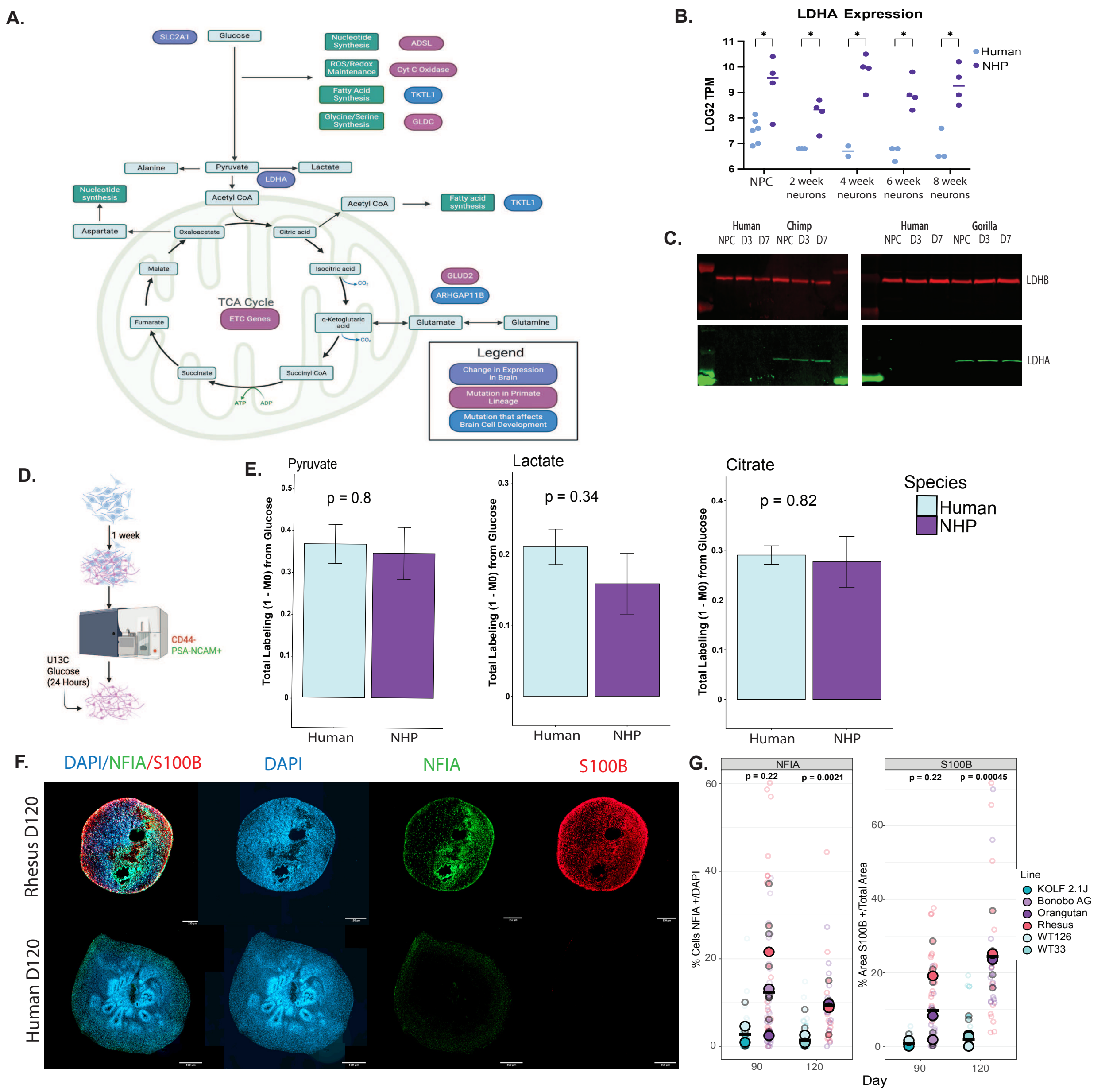

Figure 1

A) Schematic of genes that have changed in human and primate evolution. Genes in purple are those that have established gene expression differences in the brain, those in magenta have a mutation in the primate lineage, and those in blue have a mutation that is known to affect brain development. Made in *Biorender*.

B) LDHA expression in NPC and neurons over the course of differentiation, replotted from Linker et al. (2022). Each dot represents a sample. P-value < 0.05 = *.

C) Western blot of LDHA protein expression over the course of neuronal differentiation. Cells were collected at the neural progenitor cell (NPC), day 3 post-differentiation, and day 7 post-differentiation. Protein collected from two human lines, one chimp and one gorilla line. LDHB serves as the control.

D) Schematic of collection for metabolic data from neurons.

E) Human and non-human primate (NHP) tracing data from glucose to lactate, pyruvate and citrate. Technical replicates are averaged together (3 per line). Error bars are standard error of the mean. Y-axis shows what fraction of the given metabolite is labeled from [U-$^{13}C_6$] glucose. N= 5 cell lines for the NHP group, N = 3 lines for the human group. Student's t-test was used.

F) Representative images from 120-day old organoids from human and rhesus macaque. DAPI staining in blue, NFIA in green, S100B in red. Scale bar of length 150 $\mu$m.

G) Quantification of organoids at day 90 and day 120 for NFIA and S100B. Large circles demonstrate averages per line. Filled in smaller circles indicate organoid averages. Small circles unfilled are individual data points. Quantification done for 4- 5 points in each organoid, for 4-5 organoids per line. Humans are in blue, great apes are in purple, and rhesus macaque (old world monkey) are in red. Y-axis indicates the percent of cells (left) or percent of image area (right) positive for the given marker. Statistics performed on line (biological replicate) averages. Student's t-test was used.

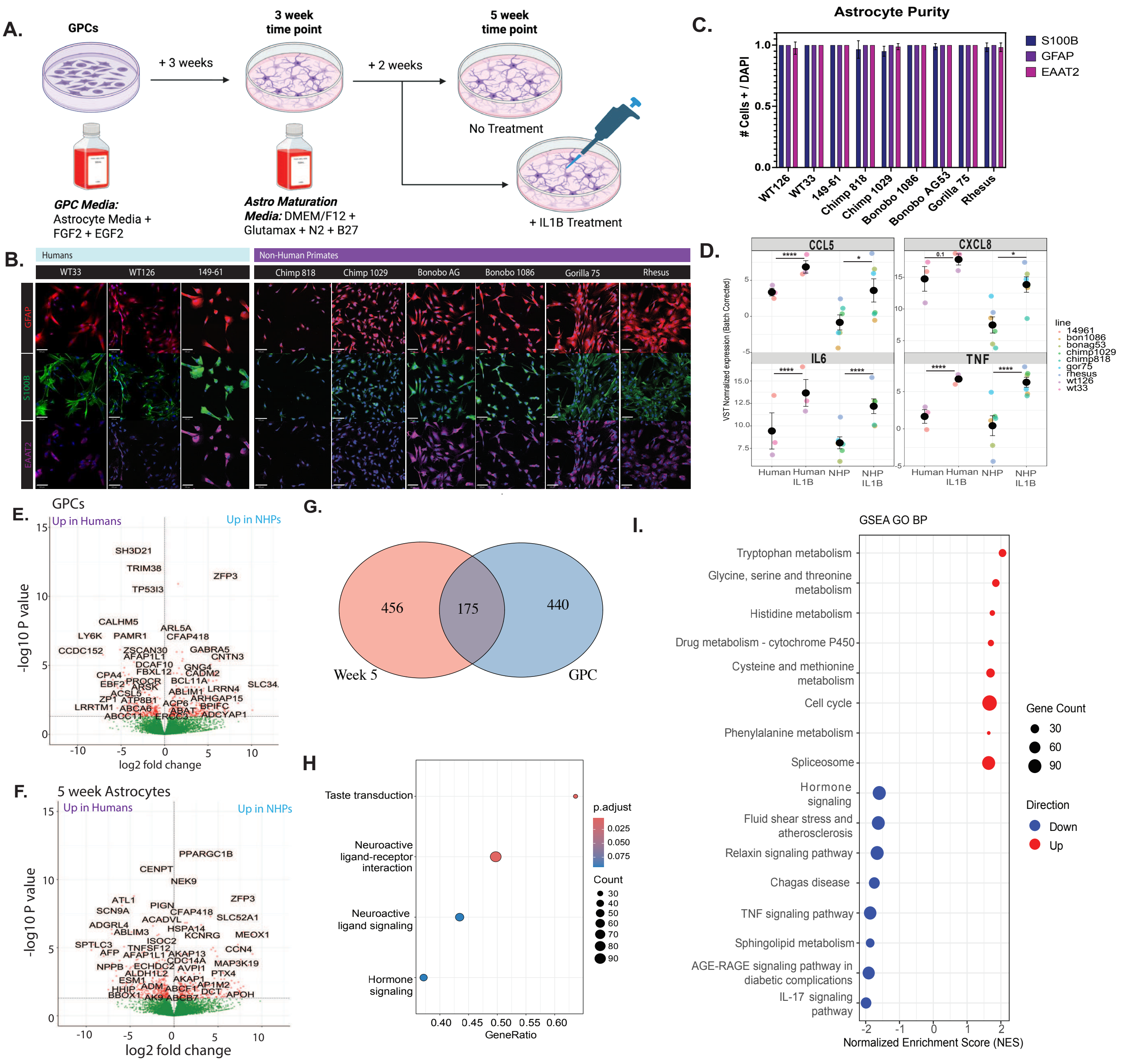

Figure 2

A) Schematic of RNA sequencing collection and differentiation of astrocytes, as well as IL-1β treatment as shown in Figure 2D.

B) Staining for key astrocyte markers in all our astrocyte lines. Green shows S100B staining, magenta shows EAAT2 staining, and red shows GFAP staining.

C) Quantification of staining as shown in Figure 2B. Each image was divided into quadrants and about 20 cells were counted for the presence or absence of the given marker. Y-axis shows the number of cells that were positive/DAPI averaged over the four quadrants. Error bars show standard deviation.

D) Plot showing the changes in expression of key cytokines and inflammatory-responsive genes in response to IL-1β treatment. Four key genes are shown. Each data point is the average of technical replicates for each line, and dot color indicates line. Statistics were performed by comparing human to human + IL-1β and NHP astrocytes versus NHP astrocytes + IL-1β. Black circles indicate mean, and error bars indicate standard error. P-values are adjusted p-values from DESEQ2 with the formula ~ line + IL-1β. * = $p < 0.05$; ** = $p < 0.01$ ; *** = $p < 0.001$ ; **** = $p < 0.0001$.

E) Volcano plot of differentially expressed genes between human and NHP genes at the GPC time point. Positive LFC indicates increased expression in NHPs, negative LFC indicates increased expression in humans. P-values generated with DESEQ2 as described for Figure 2D.

F) Volcano plot of differentially expressed genes between human and NHP genes at the five-week-old differentiated astrocyte time point. Data analysis performed as described for Figure 2E.

G) Venn diagram showing differentially expressed genes between human and NHPs at the GPC stage (right) and five-week-old astrocytes (left). 175 genes are differentially expressed at both stages of maturation as determined by DESEQ2.

H) GSEA for GPC RNA-sequencing data comparing human and NHP GPCs. Color shows p-value. Size of dot shows how many genes drive enrichment in the given category.

I) GSEA of five-week-old astrocyte RNA-sequencing data comparing human and NHP data in DESEQ2. Blue (negative normalized enrichment score) indicates higher expression of the pathway in human astrocytes. Red indicates higher expression of the pathway in NHP astrocytes. Dot size indicates how many genes drive enrichment in the given category.

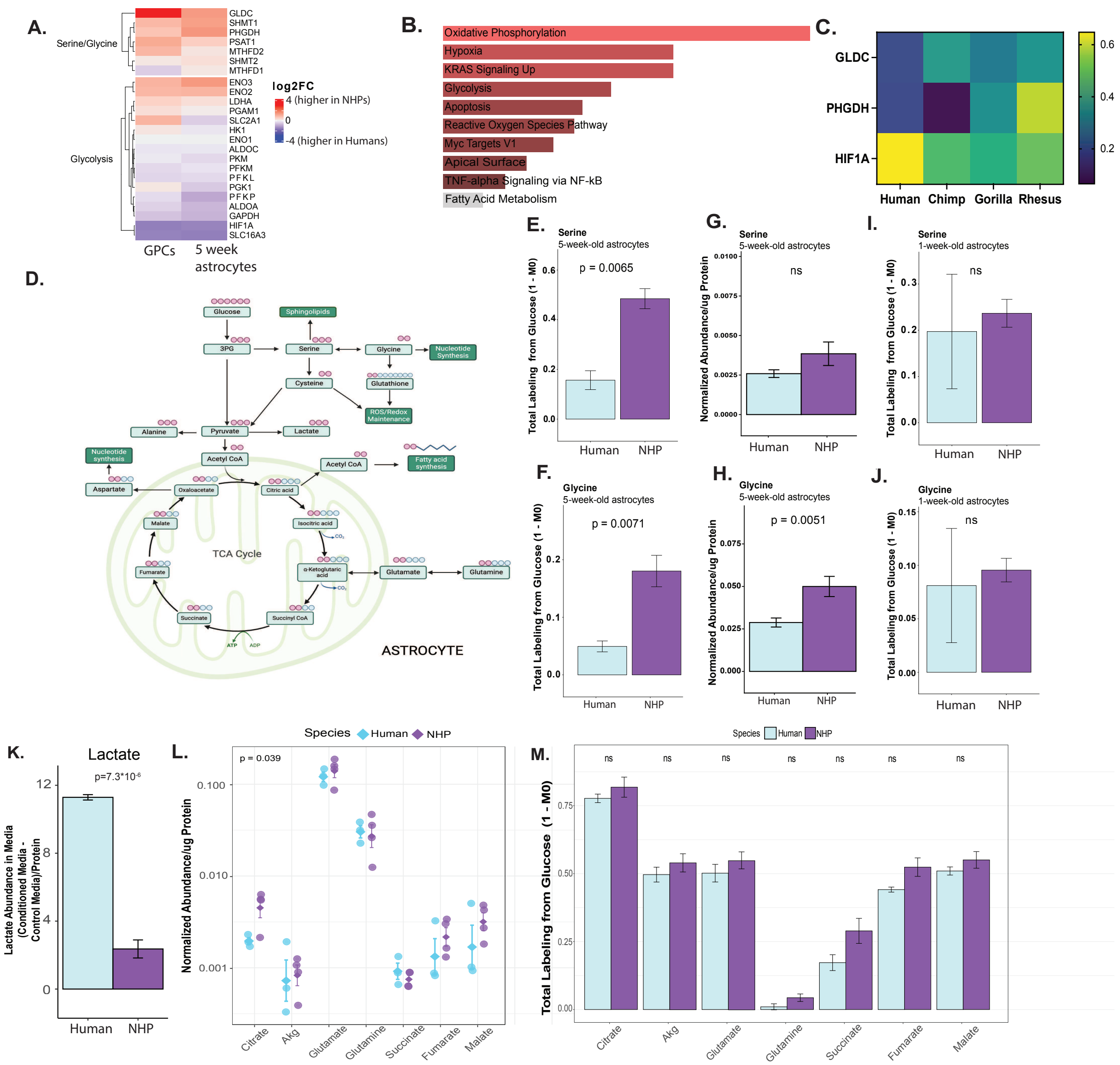

Figure 3

A) Expression of key metabolic genes at the GPC and five-week time points. Color indicates LOG2FC, with a positive log2 fold change indicating higher expression in NHP astrocytes compared to human astrocytes and a negative value indicating higher expression in human astrocytes. LFC values generated with DESEQ2.

B) MSigDB Hallmark enrichment for genes differentially expressed between human and chimpanzee astrocyte clusters in post-mortem data. Differential expression values and data used from Jorstad et al. (2023). Analysis done with Enrichr using MSigDB Hallmark 2020 Analysis. Red coloring indicates unadjusted p-value < 0.05.

C) Average expression of key genes in astrocyte clusters from sn-RNAseq data in different species (re-graphed from Jorstad et al., 2023 and Cytosplore Viewer).

D) Schematic of [U-$^{13}C_6$] glucose carbon labeling experiments. Red and blue circles indicate carbons. Red carbons indicate those that may end up labeled by the addition of [U-$^{13}C_6$] glucose.

E) Serine labeling from [U-$^{13}C_6$] glucose in 5-week-old astrocytes. Y axis shows 1 minus the unlabeled amount over the total amount of the given metabolite. N = 2 for human and N=5 for NHP (biological replicates). Technical replicates are averaged together. Bars show standard error. Student t-test was used.

F) Glycine labeling from [U-$^{13}C_6$] glucose trace in five-week-old astrocytes. Analysis performed as described for Figure 3E.

G) Intracellular serine abundance normalized to protein amount and an internal standard in five-week old astrocytes. Analysis performed as described for Figure 3E.

H) Intracellular glycine abundance normalized to protein amount and an internal standard in five-week old astrocytes. Analysis performed as described for Figure 3E.

I) Serine labeling from [U-$^{13}C_6$] glucose trace in one-week-old astrocytes. Y axis shows 1 minus the unlabeled amount over the total amount of the given metabolite. N = 2 for human and N= 5 for NHP (biological replicates). Technical replicates are averaged together. Bars show standard error. Student t-test was used.

J) Glycine labeling from [U-$^{13}C_6$] glucose trace in one-week-old astrocytes. Analysis performed as described for Figure 3I.

K) Lactate abundance in media in human versus NHP astrocyte samples. Bars denote standard error. Values normalized to baseline media (unconditioned) abundances. Technical replicates run in triplicate and averaged together per biological replicate (line). Biological replicates: N = 2 for human and N=6 for NHPs. Bars show standard error. Student's t-test was performed.

L) Normalized abundance of TCA cycle intermediates and offshoot metabolites measured intracellularly using a GC-MS. Values normalized to norvaline, an internal control, and the amount of protein. Technical replicates averaged together; biological replicates are individual lines. N = 2 for human and N=5 for NHPs. Y-axis is log scale. Bars show standard error. Student's t-test was performed.

M) TCA cycle labeling from [U-$^{13}C_6$] glucose in 5-week-old astrocytes. Y axis shows 1 minus the unlabeled amount over the total amount of the given metabolite. N = 2 for human and N = 5 for NHP (biological replicates). $P < 0.05$ is labeled. Bars show standard error. Student's t-test was performed.

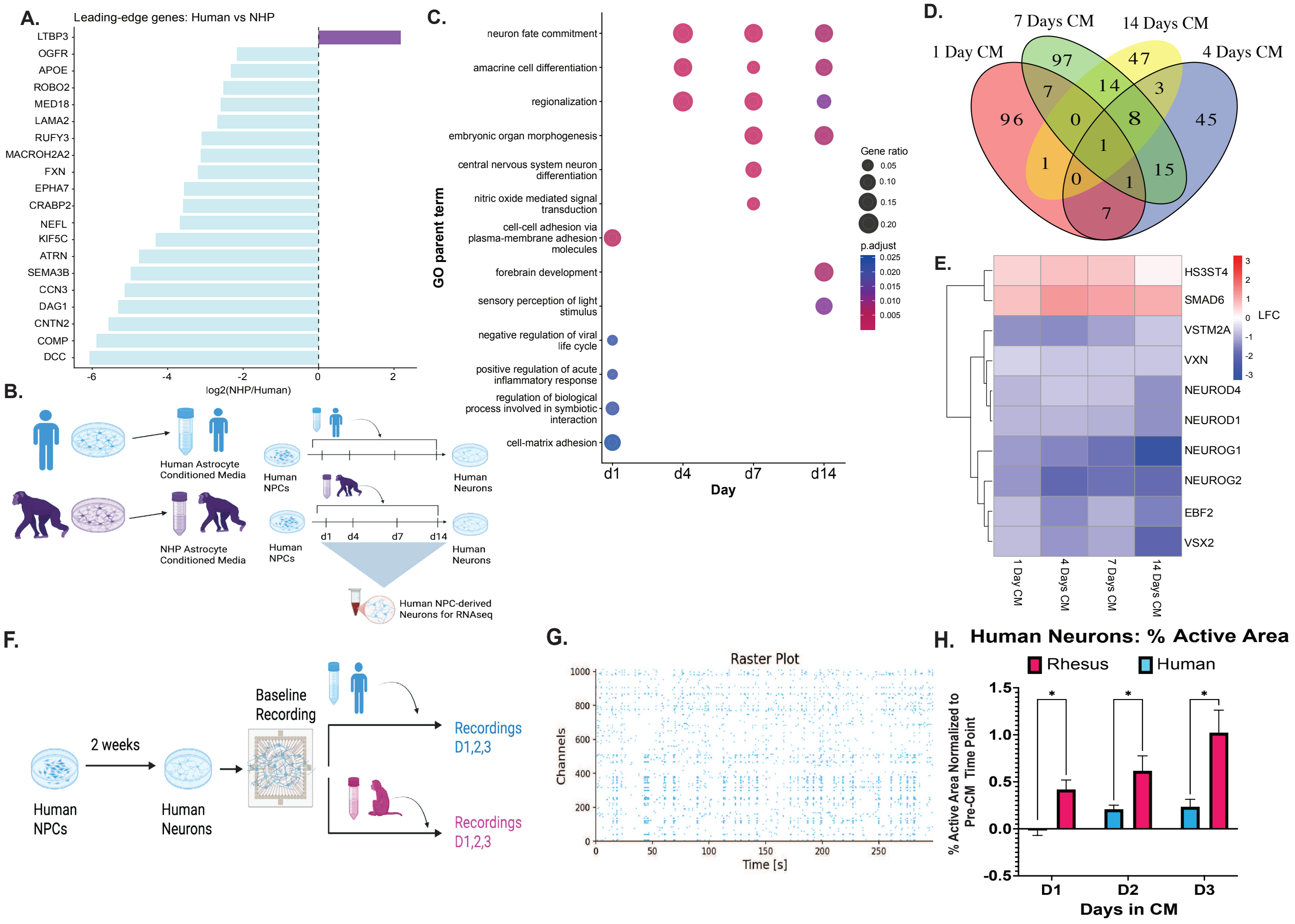

Figure 4

A) Leading edge proteins from secretome of human versus NHPs for top GSEA categories. Astrocyte secreted proteins more abundant in human samples are in light blue, and those more abundant in NHP samples are in purple. N = 3 lines (2 technical replicates per line) for human and 4 for NHP lines (2 technical replicates per line).

B) Schematic of conditioned media experiments. Neuronal differentiation media was added to human or NHP astrocytes and then collected, filtered, and added to human NPCs. Three human astrocyte lines were used and four NHP lines were used.

C) GO term analysis from RNA-sequencing from human neurons cultured with human versus NHP astrocyte conditioned media. Dot colors indicate adjusted p-value for enrichment. Gene ratio indicates the number of genes in the given category that are significantly differentially expressed. X-axis indicates the days in conditioned media before the NPC-derived neurons were collected for RNA.

D) Venn diagram of differentially expressed genes (FDR < 0.1) in neurons between human and NHP astrocyte conditioned media treatment for each time point (duration in conditioned media).

E) Genes that were differentially expressed (FDR <0.1) in three out of four of the conditions were plotted. Color indicates log fold change between human conditioned media neurons and NHP conditioned media neurons. Red indicates higher expression of the given gene in NHP astrocyte conditioned media condition, blue indicates higher expression in human astrocyte conditioned media condition. LFC shown for each duration of time in conditioned media (1 day in CM, 4 days, 7 days, or 14 days).

F) Schematic for MEA experiments. Human and rhesus astrocyte conditioned media collected and added to two-week-old NPC-derived neurons seeded onto an MEA plate. Recordings normalized to baseline recording prior to addition of any astrocyte conditioned media.

G) Representative trace of firing activity for multi-electrode array assay. The y-axis indicates different channels of electrical activity recorded, and the x-axis represents the time in the recording (from 0 to 300 seconds).

H) Plot of the percent area of a well with activity during an activity scan. Each well was normalized to its own pre-conditioned media recording time point. Blue bars indicate wells of human neurons fed human astrocyte conditioned media. Red indicates wells of human neurons fed rhesus astrocyte conditioned media. P-values indicate differences between human and rhesus activity at each time point. X-axis shows days in conditioned media. Neurons were differentiated for two weeks and replated on MEA plate before the addition of species-specific conditioned media. N = 5 for human conditioned media and N=5 for rhesus. Error bars show standard error. Asterisks indicate $p < 0.05$. Student's t-test was used. Two human lines and one rhesus macaque line were used for astrocytes. Replicates were treated as different wells of the MEA plate.

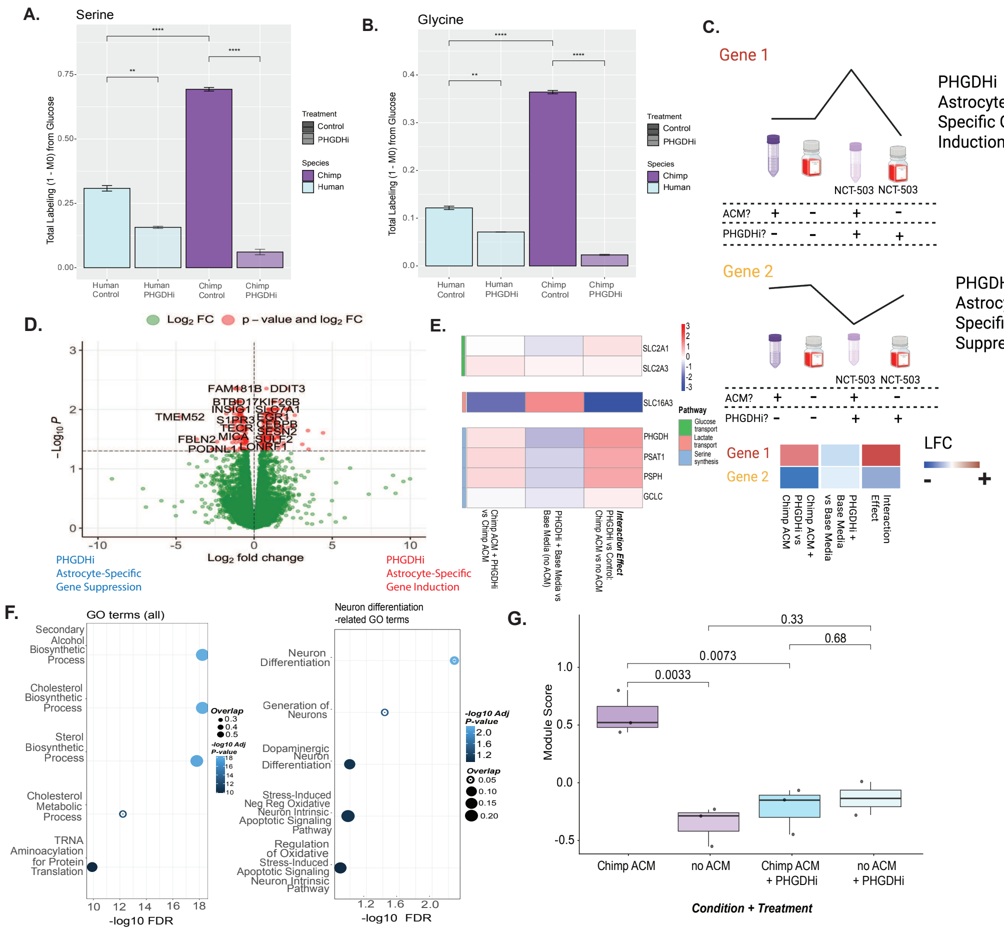

Figure 5

A) Serine labeling from [U-$^{13}C_6$] glucose in 5-week-old astrocytes treated with a PHGDH inhibitor. Y axis shows 1 minus the unlabeled amount over the total amount of the given metabolite. Bars show standard error. * = $p < 0.05$; ** = $p < 0.01$ ; *** = $p < 0.001$ ; **** = $p < 0.0001$. Purple indicates chimpanzee astrocyte media, and light purple shows media from chimp astrocytes treated with a PHGDH inhibitor (NCT-503) for 24 hours. Blue indicates human astrocyte media, and lighter blue shows media from human astrocytes treated with a PHGDH inhibitor. Student's t-test was used. One human and one chimpanzee line was used. Replicates are different wells.

B) As described for Figure 5A, but for glycine.

C) Schematic of PHGDH inhibition RNA-sequencing experiments. Representative heatmap is shown below (not real data).

D) Volcano plot of genes differentially regulated in response to PHGDH inhibition in chimpanzee astrocytes. DESEQ2 formula of treatment + conditioned media + treatment: conditioned media was used. Positive LFC indicates genes that show increases in gene expression specifically due to the effect of PHGDH inhibition on the chimp astrocytes. Negative LFC values indicate genes that are downregulated. One chimpanzee line and one human line were used, and different wells were treated as replicates.

E) Key metabolic gene expression in neurons in response to PHGDHi in chimp astrocytes. LFC is indicated in the colors. On the x-axis, LFC is shown for, respectively, comparing the expression in neurons exposed to chimp astrocyte conditioned media with PHGDH inhibition vs regular chimp astrocyte conditioned media; just the PHGDH inhibitor in the base neuronal media versus base neuronal media without inhibitor; and the interaction effect of PHGDH inhibition with the astrocyte conditioned media. Key genes were picked based on their involvement in metabolic pathways that changed in astrocytes (see Figure 3).

F) GO analysis of genes differentially regulated due to the effect of PHGDH inhibition on the chimp astrocytes. DESEQ2 formula of treatment + conditioned media + treatment: conditioned media was used. Left side shows top enriched GO terms overall for differentially expressed genes, with size of the dot indicating the amount of overlap with all the terms in the category. X-axis shows -log10 of FDR and color of dot indicates -log10 p-value. Top 5 terms are shown. The graph on the right shows the top significantly enriched GO terms related to neuronal functioning.

G) Module score for differentially expressed genes between human and NHP media conditions at one time point (the day four time point) when applied to the PHGDH inhibitor experiment. X-axis shows neurons in different conditions (chimp astrocyte conditioned media, regular base media, chimp astrocyte conditioned media from chimp astrocytes treated with PHGDH inhibitor, and regular base media with PHGDH inhibitor).

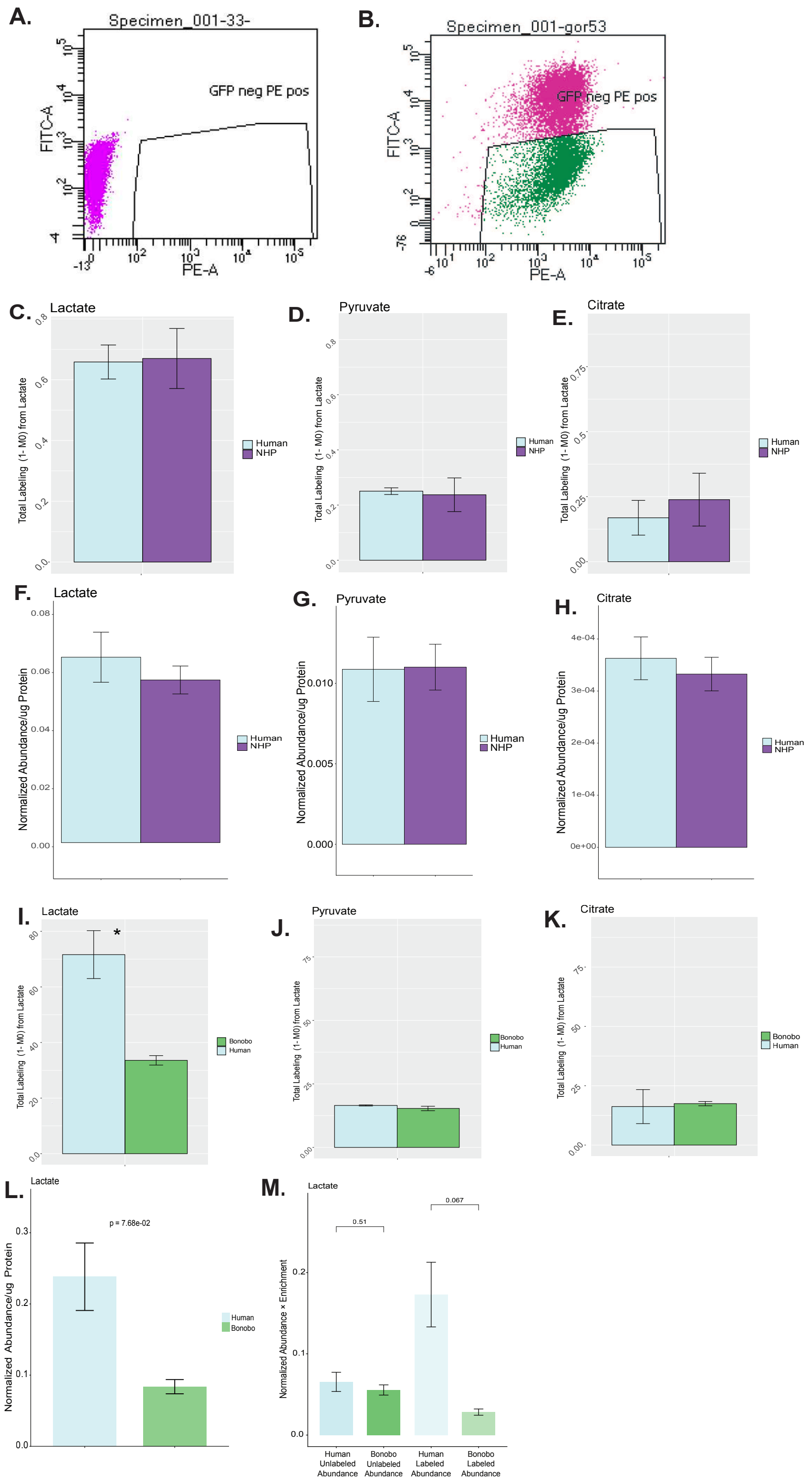

Supplemental Figure 1

A) Example FACS gating from an unstained control human NPC line.

B) Example FACS gating for PSA-NCAM + and CD44 - cells from one gorilla NPC line.

C) [U-$^{13}C_3$] lactate tracing in one-week-old sorted neurons from three human and four NHP lines. Bars denote standard error. Technical replicates run in triplicate and averaged together per biological replicate (line). Y-axis shows 1 - M0 (or total labeling from lactate). Student's t-test was used.

D) Pyruvate labeling from lactate trace, as described for Supplemental Figure 1C.

E) Citrate labeling from lactate trace, as described for Supplemental Figure 1C.

F) Intracellular abundance of lactate normalized to norvaline (internal standard) and protein as measured by BCA from lactate trace described in Supplemental Figure 1F.

G) Intracellular abundance of pyruvate, as described for Supplemental Figure 1F.

H) Intracellular abundance of citrate, as described for Supplemental Figure 1F.

I) [3-$^{13}C_1$] lactate tracing in three-week-old sorted neurons from one human and one bonobo line. Bars denote standard error. Replicates run in triplicate and represent different wells of tracing. Y-axis shows 1 - M0 (or total labeling from lactate). * = $p < 0.05$. Student's t-test was used.

J) Pyruvate labeling from lactate trace, as described for Supplemental Figure 1I.

K) Citrate labeling from lactate trace, as described for Supplemental Figure 1I.

L) Intracellular abundance of lactate normalized to norvaline (internal standard) and protein as measured by BCA from lactate trace described in Supplemental Figure 1I. P-value indicated.

M) Abundance x enrichment values (or unlabeled and labeled abundance) for data described in Supplemental Figure 1I. Student's t-test performed comparing human and bonobo unlabeled abundances and human versus bonobo labeled abundances.

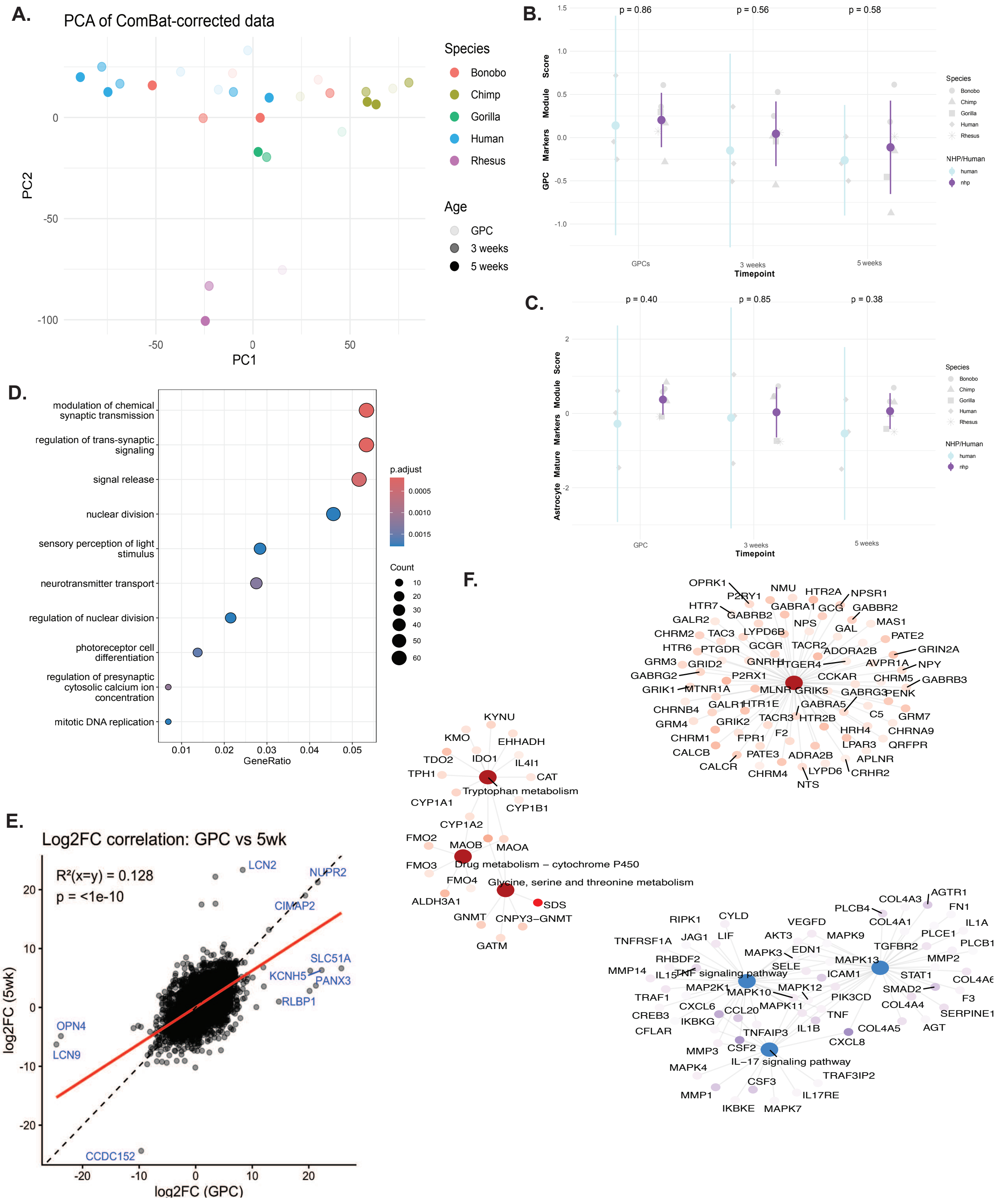

A.
PCA of ComBat-corrected data
PC1
PC2
Species
Bonobo
Chimp
Gorilla
Human
Rhesus
Age
GPC
3 weeks
5 weeks
B.
p = 0.86
p = 0.56
p = 0.58
GPC Markers Module Score
GPCs
3 weeks
5 weeks
Timepoint
NHP/Human
human
nhp
C.
p = 0.40
p = 0.85
p = 0.38
Astrocyte Mature Markers Module Score
GPC
3 weeks
5 weeks
Timepoint
D.
modulation of chemical synaptic transmission
regulation of trans-synaptic signaling
signal release
nuclear division
sensory perception of light stimulus
neurotransmitter transport
regulation of nuclear division
photoreceptor cell differentiation
regulation of presynaptic cytosolic calcium ion concentration
mitotic DNA replication
GeneRatio
p.adjust
Count
E.
Log2FC correlation: GPC vs 5wk
R²(x=y) = 0.128
p = <1e-10
log2FC (GPC)
log2FC (5wk)
LCN2
NUPR2
CIMAP2
SLC51A
KCNH5
PANX3
RLBP1
OPN4
LCN9
CCDC152
F.
Tryptophan metabolism
Drug metabolism – cytochrome P450
Glycine, serine and threonine metabolism
TNF signaling pathway
IL−17 signaling pathway

Supplemental Figure 2

A) PCA plot of human and NHP samples as GPCs, three-week-old astrocytes and five-week-old astrocytes. Species denote color, and degree of transparency shows weeks post-differentiation.

B) Module score for GPC markers based on Zhang et al., 2016. Lines indicate confidence intervals and large dot indicates mean. Individual lines are shown as gray dots and shape of dots indicates the species. Technical replicates are averaged together. Counts are normalized using DESEQ2, log scaled, and z-scored, and then averaged together to form a module score, as shown on the y-axis. P-values indicate difference between human and non-human primate lines at each time point using a student's t-test.

C) As described for Supplemental Figure 2B, for the mature astrocyte markers in Zhang et al., 2016.

D) GO analysis for genes that are differentially expressed between humans and NHPs in both GPCs and five-week-old astrocytes.

E) Plot of LFC values comparing human vs NHP in GPCs vs 5-week-old astrocytes. Dotted line shows x=y line. Red line shows the best fit line. $R^2$ value and P-value show fit to the given red line.

F) GSEA KEGG analysis leading edge genes within significantly enriched categories. Red gene dot indicates positive log fold change (more highly expressed in NHP five-week-old astrocytes), and blue indicates negative log fold change (more highly expressed in human five-week-old astrocytes).

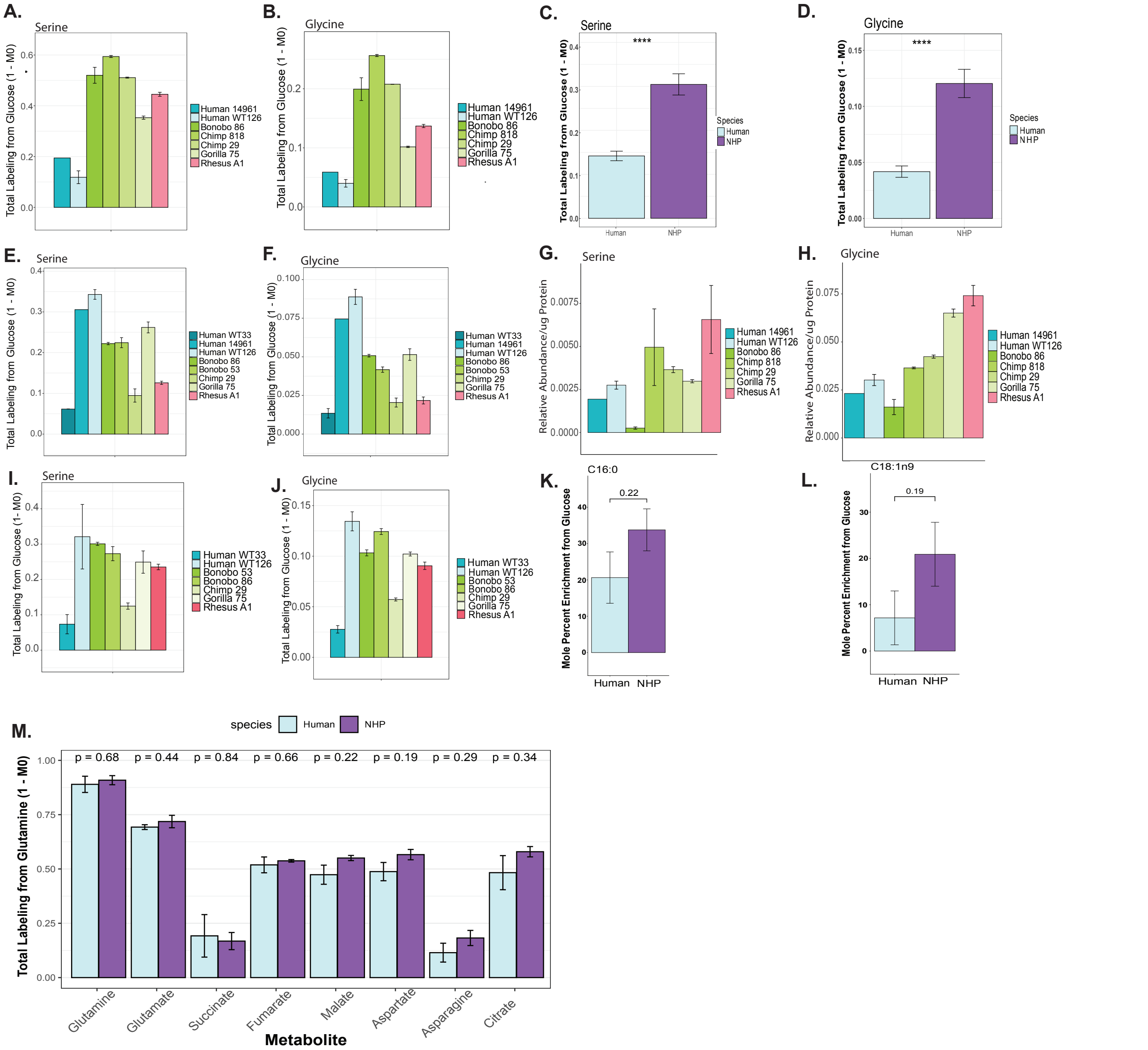

Supplemental Figure 3

A) [U-$^{13}C_6$] glucose tracing in five-week-old astrocytes for serine. Y axis shows 1 minus the unlabeled amount over the total amount of the given metabolite. X-axis separated by different lines, with 1 - 3 technical replicates per line (different wells of a plate). Blue shows human lines, green shows great apes and red shows rhesus macaque line (old world monkey). Error bars show standard error. Data as shown in Figure 3E but separated by line.

B) [U-$^{13}C_6$] glucose tracing in five-week-old astrocytes for glycine. Data as described for Supplemental Figure 3A. Data as shown in Figure 3F but separated by line.

C) Repeat experiment -- [U-$^{13}C_6$] glucose tracing in five-week-old astrocytes for serine. Y axis shows 1 minus the unlabeled amount over the total amount of the given metabolite. N = 3 for human and N=4 for NHP (biological replicates). Technical replicates are averaged together. Bars show standard error. Student t-test was used. * = $p < 0.05$; ** = $p < 0.01$ ; *** = $p < 0.001$ ; **** = $p < 0.0001$.

D) [U-$^{13}C_6$] glucose tracing in five-week-old astrocytes for glycine. Data as described for Supplemental Figure 3C.

E-F) Labeling data as described for Supplemental Figure 3A but in the media from 5-week-old-astrocytes as opposed to intracellular labeling data. E shows serine, and F shows glycine.

G-H) Intracellular abundance data for five-week-old astrocytes. Y axis shows abundance of given metabolite normalized to an internal standard (norvaline) and protein as measured by BCA. X-axis separated by different lines, with 1 - 3 technical replicates per line (different wells of a plate). Blue shows human lines, green shows great apes and red shows rhesus macaque line (old world monkey). G shows serine and H shows glycine. Bars shows standard error.

I-J) Labeling data as described for Supplemental Figure 3A-C but for one-week old astrocytes. as opposed to intracellular labeling data. I shows serine, J shows glycine.

K) Mole percent enrichment value for palmitate in human and NHP lines from [U-$^{13}C_6$] glucose tracing. Technical replicates are averaged together. N = 3 lines for human and N=4 lines for NHP. Bars show standard error. Student's t-test was used.

L) As described in K for oleic acid.

M) [U-$^{13}C_5$] glutamine labeling data from five-week-old astrocytes. Y axis shows 1 minus the unlabeled amount over the total amount of the given metabolite. Metabolites from the TCA cycle and their branch off points are shown in order of the cycle. Technical replicates are averaged together. N = 3 for human and N=4 for NHP (biological replicates). P-values show comparison between human and NHP samples for each metabolite. Bars show standard error. Student's t-test was used.

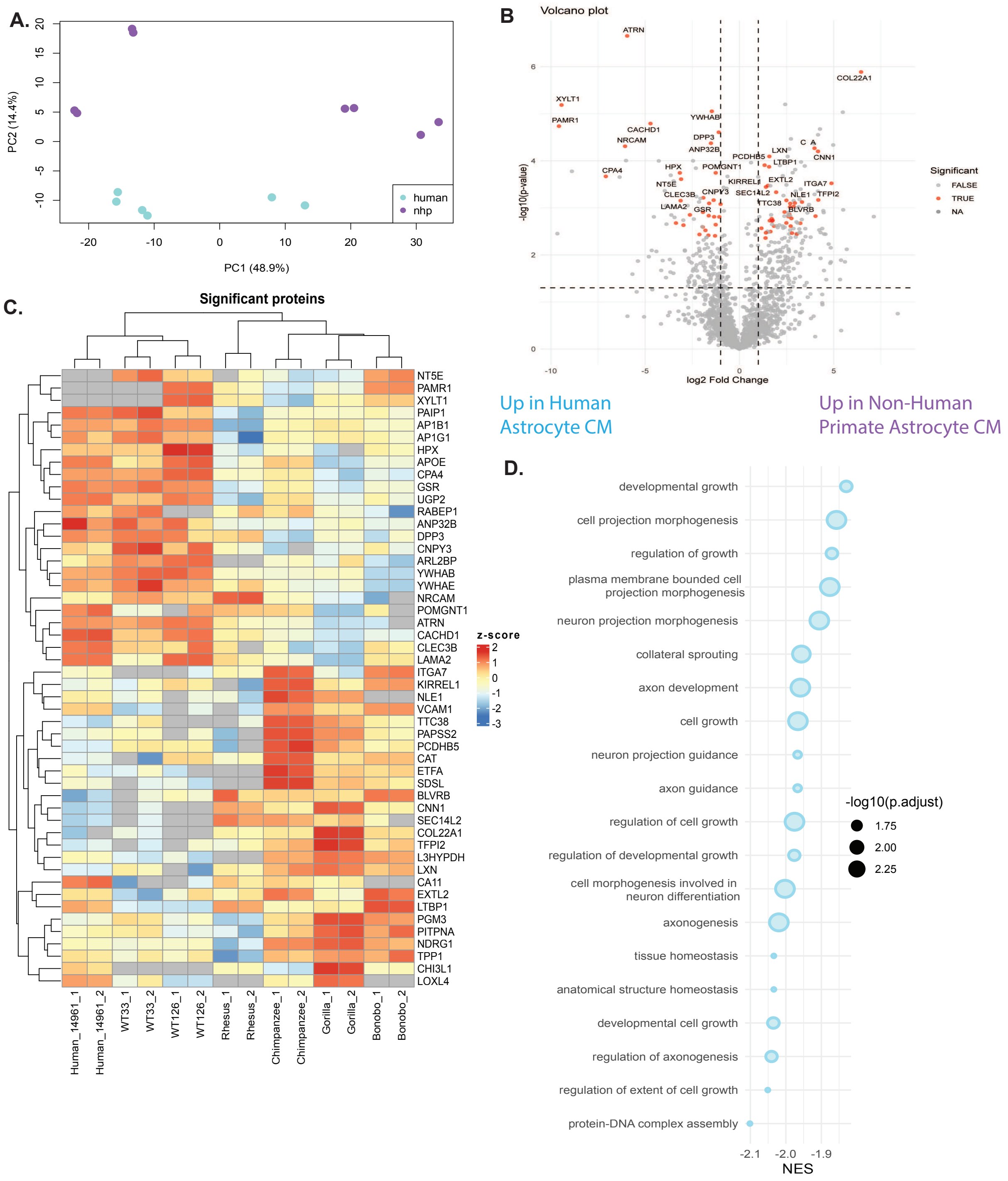

A.
PC2 (14.4%)
PC1 (48.9%)
human
nhp
B
Volcano plot
-log10(p-value)
log2 Fold Change
ATRN
COL22A1
XYLT1
PAMR1
CACHD1
NRCAM
YWHAB
DPP3
ANP32B
PCDHB5
LXN
LTBP1
CNN1
HPX
POMGNT1
CPA4
NT5E
KIRREL1
EXTL2
ITGA7
CLEC3B
CNPY3
SEC14L2
NLE1
TFPI2
TTC38
LAMA2
GSR
BLVRB
Significant
FALSE
TRUE
NA
Up in Human
Astrocyte CM
Up in Non-Human
Primate Astrocyte CM
C.
Significant proteins
z-score
NT5E
PAMR1
XYLT1
PAIP1
AP1B1
AP1G1
HPX
APOE
CPA4
GSR
UGP2
RABEP1
ANP32B
DPP3
CNPY3
ARL2BP
YWHAB
YWHAE
NRCAM
POMGNT1
ATRN
CACHD1
CLEC3B
LAMA2
ITGA7
KIRREL1
NLE1
VCAM1
TTC38
PAPSS2
PCDHB5
CAT
ETFA
SDSL
BLVRB
CNN1
SEC14L2
COL22A1
TFPI2
L3HYPDH
LXN
CA11
EXTL2
LTBP1
PGM3
PITPNA
NDRG1
TPP1
CHI3L1
LOXL4
Human_14961_1
Human_14961_2
WT33_1
WT33_2
WT126_1
WT126_2
Rhesus_1
Rhesus_2
Chimpanzee_1
Chimpanzee_2
Gorilla_1
Gorilla_2
Bonobo_1
Bonobo_2
D.
developmental growth
cell projection morphogenesis
regulation of growth
plasma membrane bounded cell projection morphogenesis
neuron projection morphogenesis
collateral sprouting
axon development
cell growth
neuron projection guidance
axon guidance
regulation of cell growth
regulation of developmental growth
cell morphogenesis involved in neuron differentiation
axonogenesis
tissue homeostasis
anatomical structure homeostasis
developmental cell growth
regulation of axonogenesis
regulation of extent of cell growth
protein-DNA complex assembly
-log10(p.adjust)
1.75
2.00
2.25
NES

Supplemental Figure 4

A) PCA of secretomics of human and NHP astrocytes. 3 biological replicates for human (lines) and four biological replicates for NHPs. 2 technical replicates per line.

B) Volcano plot of differentially secreted proteins in humans versus NHPs. Technical replicates treated as such in MSstats in R. abs(LOG2FC) > 1 and p.adjust < 0.05 and missingPercentage < 0.3 used as cutoffs. LFC < 0 (left side) higher in abundance in human samples, LFC > 0 higher in abundance in NHP samples.

C) Heatmap of top differentially secreted proteins sorted by p.adjust. Grey boxes indicate missing values. Proteins with missingPercentage > 0.3 excluded.

D) GSEA for human versus NHP secretome. Top significantly differentially enriched pathways included. Pathways enriched in human secretome are colored in light blue (NES < 0).

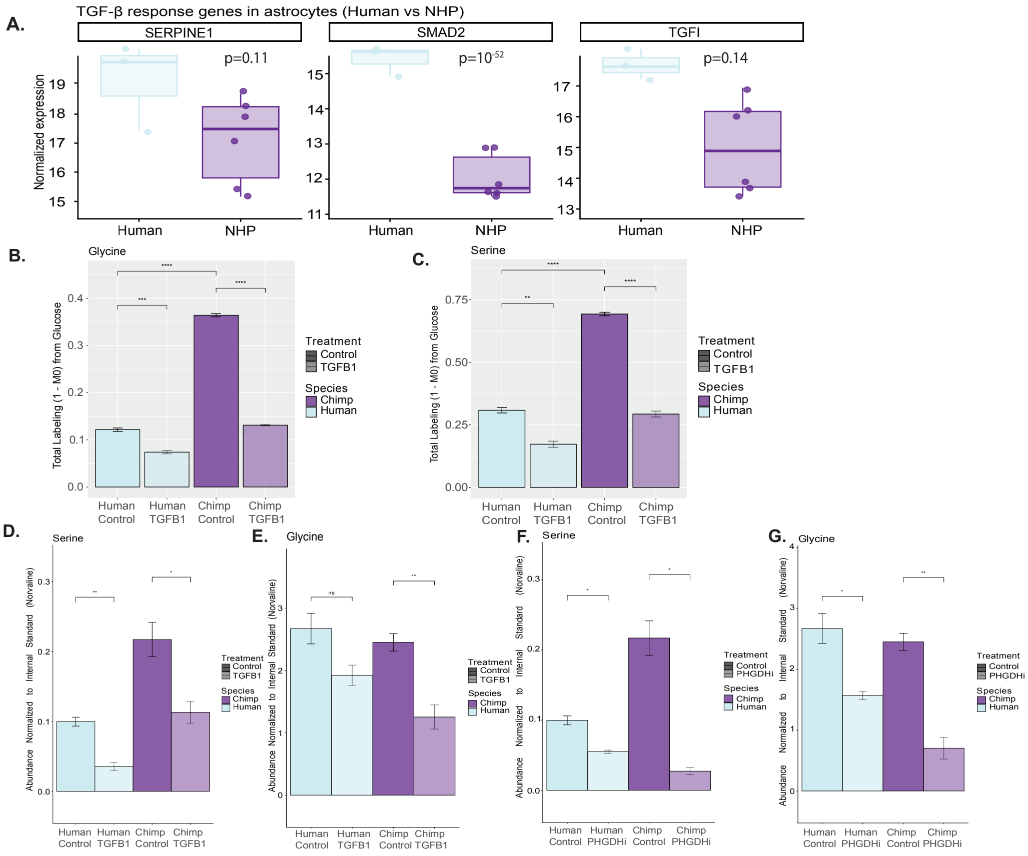

Supplemental Figure 5

A) VST normalized and batch corrected counts for TGFβ response genes. P-values as reported from DESEQ2.

B) Glycine labeling from [U-$^{13}C_6$] glucose. Y axis shows 1 minus the unlabeled amount over the total amount of the given metabolite. Purple indicates chimpanzee astrocyte media, and light purple shows media from chimp astrocytes treated with TGFβ1. Blue indicates human astrocyte media, and lighter blue shows media from human astrocytes treated with TGFβ1. Three wells (replicates) per condition, one line per species was used. Error bars show standard error. * = $p < 0.05$; ** = $p < 0.01$ ; *** = $p < 0.001$ ; **** = $p < 0.0001$. Student's t-test was used.

C) As described for Supplemental Figure 5B for serine labeling.

D) Intracellular abundance of serine normalized to norvaline (internal standard) from glucose trace described in Supplemental Figure 5C. Purple indicates chimpanzee astrocyte media, and light purple shows media from chimp astrocytes treated with TGFβ1. Blue indicates human astrocyte media, and lighter blue shows media from human astrocytes treated with TGFβ1. Three wells (replicates) per condition, one line per species was used. Error bars show standard error. * = $p < 0.05$; ** = $p < 0.01$ ; *** = $p < 0.001$ ; **** = $p < 0.0001$. Student's t-test was used.

E) As described for Supplemental Figure 5D for glycine.

F) Intracellular abundance of serine normalized to norvaline (internal standard) from glucose trace described in Figure 5B. Purple indicates chimpanzee astrocyte media, and light purple shows media from chimp astrocytes treated with PHGDHi. Blue indicates human astrocyte media, and lighter blue shows media from human astrocytes treated with PHGDHi. Three wells (replicates) per condition, one line per species was used.

G) As described for Supplemental Figure 5F for glycine.
